# Partial rescue of V1V2 mutant infectivity by HIV-1 cell-cell transmission supports the domain’s exceptional capacity for sequence variation

**DOI:** 10.1186/s12977-014-0075-y

**Published:** 2014-09-25

**Authors:** Oliver F Brandenberg, Peter Rusert, Carsten Magnus, Jacqueline Weber, Jürg Böni, Huldrych F Günthard, Roland R Regoes, Alexandra Trkola

**Affiliations:** Institute of Medical Virology, University of Zurich, Zurich, Switzerland; University Hospital Zurich, Division of Infectious Diseases, Zurich, Switzerland; Institute of Integrative Biology, ETH Zurich, Zurich, Switzerland

**Keywords:** HIV, Cell-cell transmission, V1V2, Entry, Neutralization, Antibody escape

## Abstract

**Background:**

Variable loops 1 and 2 (V1V2) of the HIV-1 envelope glycoprotein gp120 perform two key functions: ensuring envelope trimer entry competence and shielding against neutralizing antibodies. While preserving entry functionality would suggest a high need for V1V2 sequence optimization and conservation, shielding efficacy is known to depend on a high flexibility of V1V2 giving rise to its substantial sequence variability. How entry competence of the trimer is maintained despite the continuous emergence of antibody escape mutations within V1V2 has not been resolved. Since HIV cell-cell transmission is considered a highly effective means of virus dissemination, we investigated whether cell-cell transmission may serve to enhance infectivity of V1V2 variants with debilitated free virus entry.

**Results:**

In a detailed comparison of wt and V1V2 mutant envelopes, V1V2 proved to be a key factor in ascertaining free virus infectivity, with V1V2 mutants displaying significantly reduced trimer integrity. Despite these defects, cell-cell transmission was able to partially rescue infectivity of V1V2 mutant viruses. We identified two regions, encompassing amino acids 156 to 160 (targeted by broadly neutralizing antibodies) and 175 to 180 (encompassing the α4β7 binding site) which were particularly prone to free virus infectivity loss upon mutation but maintained infectivity in cell-cell transmission. Of note, V1V2 antibody shielding proved important during both free virus infection and cell-cell transmission.

**Conclusions:**

Based on our data we propose a model for V1V2 evolution that centers on cell-cell transmission as a salvage pathway for virus replication. Escape from antibody neutralization may frequently result in V1V2 mutations that reduce free virus infectivity. Cell-cell transmission could provide these escape viruses with sufficiently high replication levels that enable selection of compensatory mutations, thereby restoring free virus infectivity while ensuring antibody escape. Thus, our study highlights the need to factor in cell-cell transmission when considering neutralization escape pathways of HIV-1.

**Electronic supplementary material:**

The online version of this article (doi:10.1186/s12977-014-0075-y) contains supplementary material, which is available to authorized users.

## Background

The HIV-1 envelope trimer, consisting of three gp120 – gp41 heterodimers, is responsible for mediating HIV entry into target cells and is the sole target of neutralizing antibodies elicited in HIV infected individuals [1,2]. The variable loops 1 and 2 (V1V2) of gp120 are a key component in shaping the envelope’s susceptibility to neutralization and have long been known to potently shield the trimer against antibody attack [3-13]. Mutations triggered by antibody escape are regarded as the driving force of the high intra- and inter-patient sequence variation of the V1V2 domain [14,15]. Despite its seemingly high adaptability, mutations within V1V2 can impair envelope trimer functionality and integrity as the domain mediates intra- and inter-gp120 subunit interactions at the apex of the envelope spike [16-18]. Numerous naturally occurring V1V2 mutations as well as deletion of the entire domain were described to either obliterate or strongly reduce virus infectivity [3,9,19-25]. Besides its role for trimer integrity, the V1V2 domain prevents premature adoption of the CD4-bound trimer conformation [16-18,26,27]. This is of importance for preserving a metastable trimer structure that upon receptor binding and structural rearrangements provides the energy required to complete the entry process [1,28,29]. Secondly, the closed, non-CD4 triggered trimer conformation secures shielding of neutralization-sensitive domains from antibody attack [3-13]. The epitopes profiting most from V1V2 shielding include the V3 loop, situated beneath V1V2 in the intact trimer, the CD4 binding site and CD4-induced epitopes important for co-receptor binding [3-13]. In summary, the available data show that disturbance of the quaternary orientation of V1V2 and V3 at the spike apex, either due to deletion of V1V2 or mutations within or outside V1V2, result in high increases in neutralization sensitivity to antibodies directed against these epitopes. In parallel, mutations leading to V1V2-induced transitions from a closed to an open trimer configuration result in reductions of virus entry capacity and trimer stability [3-13,19-25].

Contrasting its role in shielding against neutralizing antibodies, the V1V2 domain itself is a target of the neutralization response. Despite the high genetic variability of V1V2, certain sequence motifs are highly conserved across strains and are targeted by broadly neutralizing antibodies (bnAbs) (reviewed in [30]). These bnAb epitopes, including those of PG9 and PG16, CH01 to CH04, and PGT141 to PGT145, are formed by amino acid residues in V1V2, glycans originating from V1V2 and, in some cases, parts of V3 [31-34]. The importance of V1V2 as a potential vaccine target was recently underscored by the results of the RV144 vaccine trial, with antibodies directed against V1V2 being the main correlate of protection [35-38]. However, due to the low constraints on V1V2 sequence conservation, evidenced by the high frequency of single point mutations, length polymorphisms and changes in glycosylation pattern, escape to neutralizing antibodies directed against V1V2 or domains shielded by V1V2 develops rapidly [10,24,39-48]. A better understanding of the escape pathways that allow V1V2 to steer neutralization sensitivity and their consequences for viral infectivity and transmission is thus pivotal to support and guide vaccine design.

Here, we describe a detailed analysis of functional properties of V1V2 during the HIV-1 entry process. Specifically, we explored whether cell-cell transmission may be a potential rescue path for viruses with low entry capacity due to alterations in V1V2. Infectivity during cell-cell transmission has been estimated to be several orders of magnitude higher than free virus infection [49-54] and can overcome barriers to free virus infection including low target cell infectability, low virus production in infected cells, or low virus stability [55-57]. Due to its dual roles of shielding against neutralization and ensuring trimer integrity, resistance evolution of V1V2 is likely an iterative process that frequently generates virus variants with decreased replication capacity that require compensating mutations to thrive. Hence, we hypothesized that if emerging V1V2 mutants retain partial cell-cell transmission capacity, this would enable the virus to maintain debilitating resistance mutations while sampling compensatory mutations to restore free virus infectivity. Utilizing cell-cell transmissibility would indeed be doubly beneficial for the virus as it may not only boost infectivity but also provide a sheltered environment largely refractory to antibody neutralization [49,58,59]. As we show here, cell-cell transmissibility is indeed better preserved than free virus infectivity among naturally occurring and engineered V1V2 mutant envelopes. Hence, cell-cell transmission may constitute an important salvage pathway for replication of antibody escape variants, highlighting the need to factor in cell-cell transmission when considering antibody escape pathways of HIV-1.

## Results

### Dissecting the influence of the V1V2 domain during different virus entry pathways

Virions produced by an HIV-infected cell can infect distant target cells as free virus or transfer directly to a neighboring cell via formation of a virological synapse, the canonical cell-cell transmission pathway [49,60]. Dissecting the relative contribution of the two entry modes requires assay formats that shift the entry process to one pathway while limiting or obliterating the other. As the aim of our study was to explore differences in V1V2 functionality during HIV-1 free virus infection and cell-cell transmission, we chose assay formats that (i) distinguish between the two entry modes, (ii) allow comparisons of the respective entry efficacies, and (iii) yield comparable results across a range of target and donor cells (Additional file 1). To establish and validate our assay setups we analyzed a panel of four JR-FL env variants for their cell-cell transmission and free virus entry capacity. Besides JR-FL wildtype (wt) we probed the V2 point mutant JR-FL I165P (that has no impact on free virus infectivity and served as control) and V2 point mutations L175P [11] and D180N [61], which were previously described to interfere with env structural integrity and neutralization sensitivity.

**Figure 1:**
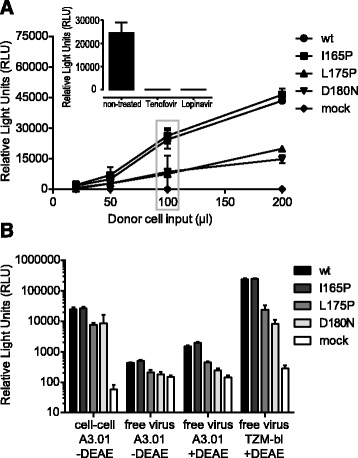
**Assessing free virus infection and cell-cell transmission with the inGLuc reporter system. (A)** Evaluation of cell-cell transmission between 293-T donor cells and A301-CCR5 target cells. The optimal input of 293-T donor cells in co-culture setups with A3.01-CCR5 target cells was determined by titration of 293-T donor cells transfected with the indicated JR-FL env variants or mock control (no env). A3.01-CCR5 target cells were kept constant at 50.000 cells. 100 μl donor cell input corresponds to approximately 15.000 293-T cells. The grey box highlights the donor cell input chosen for subsequent assays (100 μl) since this input best covered a wide range of envelope infectivities while still being in the dynamic range of the assay. Mean and SD of two independent experiments with two replicates are shown. Inset: an input of 100 μl 293-T cells transfected with JR-FL wt reporter virus was treated with the RT inhibitor Tenofovir or the protease inhibitor Lopinavir prior to starting the cell-cell co-culture. Both inhibitors completely abolished target cell infection. **(B)** Absolute GLuc reporter signals obtained in cell-cell transmission and different free virus infection setups were compared. The relative light units (RLUs) are in all cases normalized to 100 μl infectious input derived from the same donor cells, either transfected 293-T cells (cell-cell assay) or free virus stock (free virus assays). As target cells either A3.01-CCR5 cells (50.000 cells for both cell-cell and free virus assays) or TZM-bl cells (15.000 cells) were used. Mean and SD of two independent experiments with 2 replicates are shown.

To probe HIV-1 cell-cell transmission we utilized a recently introduced reporter system [56,62], which is based on co-transfection of virus producer cells with a Gaussia Luciferase reporter (inGLuc) together with plasmids encoding HIV. As described [56,62], an intron in the GLuc reporter gene and its reversed orientation in the vector prevents reporter signals from transfected donor cells or upon donor-target cell fusion (Figure 1A). Gaussia Luciferase is only produced in target cells upon successful infection by viruses that contain the inGLuc reporter and that entered either as free virus or via cell-cell transmission (Figure 1A). To monitor genuine cell-cell transmission we restricted free virus infection by omitting DEAE dextran in the co-culture medium, which we previously showed to limit infection of transformed cell lines by free virus but not cell-cell transmission (Figure 1B and [58]). Free virus infectivity was assessed in parallel using single cycle virus infection assays. To assure maximal comparability between free virus infection and cell-cell transmission, we employed cells transfected with HIV encoding plasmids and the inGLuc reporter as donor cells in cell-cell transmission setups and in parallel harvested virus-containing supernatant from the same batch of donor cells to probe free virus infection (Additional file 1).

To compare efficacies of free virus infection and cell-cell transmission one can choose between two virus input standardizations: at the level of the target cell or at the level of the donor cell. We chose the latter as we were interested to explore if virions produced by a donor cell are more infectious during cell-cell transmission or free virus infection. A set number of donor cells were used as input in cell-cell transmission assays and corresponding aliquots of donor cell culture supernatant containing free virus was used as free virus input (Additional file 1). We determined the optimal input of donor cells by titration to ascertain GLuc signals within the dynamic range of the assay (Figure 1A). To perform matched comparisons of free virus and cell-cell infectivities, we harvested both donor cells and virus supernatant from a 12-well culture and adjusted both to a total volume of 1 ml, which allowed us to express cell-cell and free virus input in μl volumes throughout our further analysis (Figure 1 and Additional file 1). As an input of 100 μl donor cell suspension (15.000 cells) provided the best dynamic range for assessing cell-cell transmission (Figure 1A), we used the corresponding amount of free virus (100 μl) as maximum volume in titrations for free virus infection (Figure 1B and Additional file 1).

For direct comparison of infectivities in both transmission modes culture conditions should be as similar as possible. We thus assessed both transmission modes in medium lacking DEAE. When we compared absolute infectivities of the JR-FL env panel we found that levels of infection were considerably higher during cell-cell transmission than during free virus infection (Figure 1B). Cell-cell transmission of JR-FL wt env was 80-fold more effective than free virus infection, in agreement with previous reports [49-51,60,63]. Free virus infectivity of env mutants L175P and D180N in absence of DEAE was only marginally above mock infected control and hence proved too low to ensure accurate detection (Figure 1B). Since quantitative comparisons of cell-cell transmission and free virus infection across viruses with different infectivities under identical culture conditions was therefore not feasible, we additionally probed free virus infectivity in presence of DEAE. Although addition of DEAE improved free virus infectivity of A3.01-CCR5 cells, absolute signals were still comparatively low, with the GLuc signal for JR-FL wt being only about 10-fold above the signal from mock-infected cells. In contrast, free virus infectivity on TZM-bl cells in presence of DEAE yielded high GLuc signals in the range of A3.01-CCR5 cell-cell transmission, with GLuc signals for JR-FL wt on TZM-bl cells being 150-fold higher than on A3.01-CCR5 cells in presence of DEAE (Figure 1B). A further advantage of the TZM-bl system was its wider dynamic range (850-fold difference between the signals of JR-FL wt and mock). Most importantly however, we observed the same pattern of free virus infectivity for the different env mutants on both A3.01-CCR5 and TZM-bl cells. Thus, we had the possibility to assess free virus infectivity with either target cell line. Regarding our interest in obtaining precise estimates of free virus entry capacity of env variants over a wide range of infectivities, we chose to use TZM-bl cells in presence of DEAE for assessment of free virus entry due to the high dynamic range and robust signals obtained in this assay.

### The V1V2 domain differentially influences free virus infection and cell-cell transmission

Concerning the role of V1V2 in shaping virus transmission we were specifically interested in addressing two aspects: i) differences in the efficacy of wt and mutant viruses within a certain transmission mode, and ii) comparison of the infectivity patterns between the two transmission modes. To enable these comparisons we calculated relative infectivities in each assay format (cell-cell or free virus infection) by normalizing the obtained mutant env infectivity to the respective wt env (Additional file 2). The derived relative infectivities (% of wt) were used to compare if and in which transmission mode env variants loose or gain activity compared to wt env.

**Figure 2:**
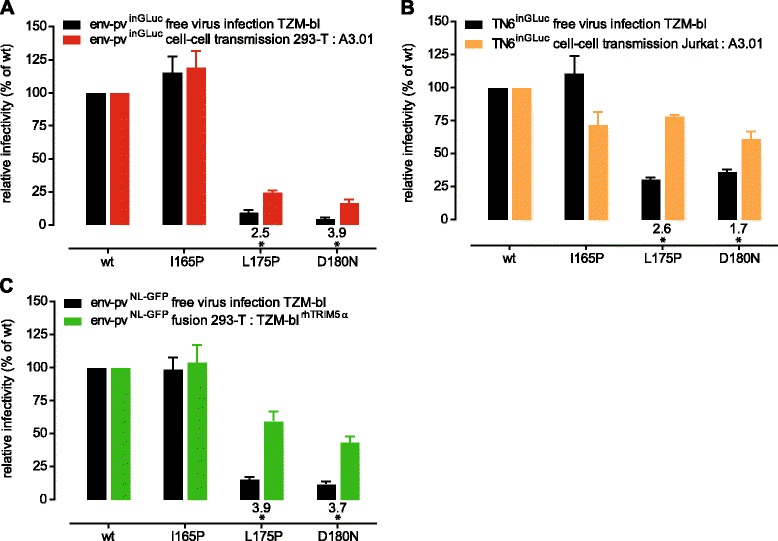
**JR-FL V2 mutations decrease cell-cell transmission capacity less than free virus infectivity. (A)** JR-FL V2 point mutant infectivities were normalized to JR-FL wt for both free virus infection (black) and cell-cell transmission (red). Values of relative efficacy of cell-cell transmission versus free virus infection are shown below the bars; a star indicates whether this difference is statistically significant as probed by multiple unpaired t-tests with alpha = 0.05. Mean and SD from 4 independent experiments performed in duplicates are depicted. **(B)** Analysis of JR-FL wt and V2 point mutants in T-cell to T-cell transmission using Jurkat donor cells (expressing replication competent TN6^inGLuc^ reporter viruses) and A301-CCR5 target cells (orange). Cell-cell transmission activity was compared to free virus infectivity of the same Jurkat derived viruses on TZM-bl cells (black). Data are normalized to JR-FL wt. Values of relative efficacy of cell-cell transmission versus free virus infection are shown below the bars; a star indicates whether this difference is statistically significant as probed by multiple unpaired t-tests with alpha = 0.05. Mean and SD from 2 independent experiments performed in duplicates are depicted. **(C)** Analysis of JR-FL wt and V2 point mutant free virus infectivity (black) and cell-cell fusion capacity (green). Data are normalized to JR-FL wt. Values of relative efficacy of cell-cell fusion versus free virus infection are shown below the bars; a star indicates whether this difference is statistically significant as probed by multiple unpaired t-tests with alpha = 0.05. Mean and SD from 2 independent experiments performed in duplicates are depicted.

Comparing the relative infectivities of the four JR-FL env variants shown in Figure 1 revealed that both cell-cell transmission and free virus infection of the L175P and D180N mutants was substantially decreased compared to wt. However, the two mutants maintained significantly higher infectivity during cell-cell transmission (2.5 and 3.9 fold higher cell-cell transmission than free virus infectivity of L175P and D180N, respectively; Figure 2A).

To ensure that the results from our cell-cell transmission assay, which utilizes 293-T cells as donor cells, were also valid for T-cell to T-cell transmission, we studied virus transmission between Jurkat (donor) and A3.01-CCR5 (target) cells (Additional file 1). Since transfection of Jurkat cells with HIV pseudotyping plasmids yielded only low levels of virus producing cells (data not shown), we transfected Jurkat cells with replication competent (rc) infectious molecular clones carrying JR-FL wt or mutant envs and the inGLuc vector (rc-TN6^inGLuc^) and assessed Jurkat to A3.01-CCR5 cell-cell transmission in absence of DEAE (Figure 2B and Additional file 1). Jurkat-derived free virus was probed in parallel for infectivity on TZM-bl cells in presence of DEAE as described. We observed the same pattern as with the 293-T donor cells, with free virus infectivity of the L175P and D180N mutants reduced to levels of 30% and 36% of wt infectivity, respectively. Cell-cell transmission capacity of the mutant envs remained at significantly higher levels with 78% and 61% of wt infectivity. This represents 2.6-fold and 1.7-fold increases in cell-cell transmission capacity over free virus infectivity for the L175P and D180N mutant, respectively (Figure 2B). Of note, the relative infectivity of the Jurkat-derived L175P and D180N mutant rc-TN6^inGLuc^ viruses on TZM-bl cells as free virus (Figure 2B) was higher compared to the 293-T derived viruses (Figure 2A) (30% versus 9.6% and 36% versus 4.2% for Jurkat and 293-T derived L175P and D180N virions, respectively), highlighting the influence of donor and target cells in virus transmission as previously suggested [56,62,64]. However, the overall infectivity pattern remained the same: regardless of the donor cells used, the relative infectivity of cell-cell transmission was higher than infectivity in free virus transmission (Figure 2A and B). Thus, since 293-T donor cells yielded results comparable to Jurkat donor cells and additionally offered the possibility to work with env pseudotyped viruses, we subsequently utilized the 293-T to A3.01-CCR5 cell-cell transmission assay for the majority of analyses.

To obtain further insight on the entry properties of mutant envs we probed their functionality during cell-cell-fusion (Figure 2C). Although cell-cell fusion is not a relevant entry mode leading to productive infection, we reasoned that assessing fusion may yield important insights on env functionality, especially for envs that are strongly impaired in both free virus infection and cell-cell transmission. Env fusion capacity and free virus infectivity were determined in parallel using a LTR-GFP reporter construct. As observed for the inGLuc reporter viruses, the L175P and D180N mutants were severely impaired in free virus infectivity (15% and 12% of wt activity for L175P and D180N, respectively; Figure 2C). This severe reduction in free virus entry capacity was not reflected in the fusion capacity of the envs, as fusogenicity was maintained at comparatively high levels (60% and 43.5% of wt activity for L175P and D180N, respectively). Since assessment of fusion capacity thus provided additional information on entry functionality, this analysis was included in our further characterization of env mutants.

### V1V2-deleted viruses lose free virus entry capacity but retain cell-cell transmission and fusion activity

**Figure 3:**
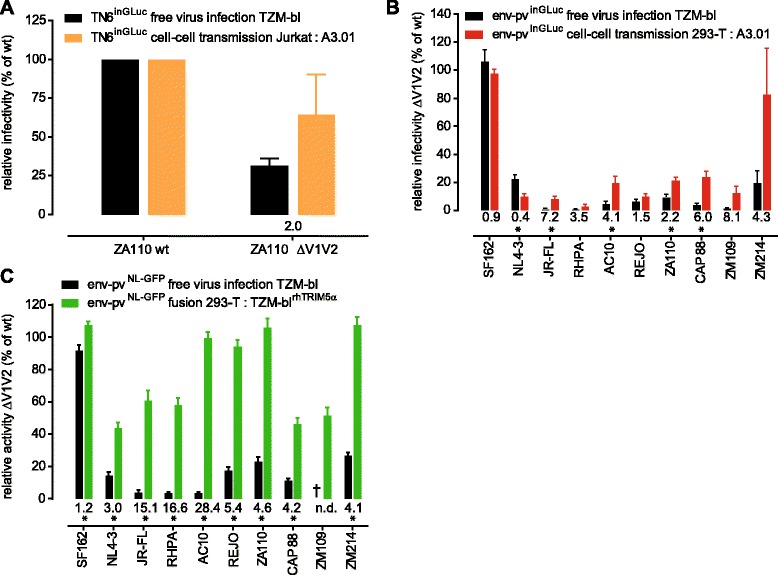
**Higher tolerance towards loss of V1V2 during cell-cell transmission and cell-cell fusion compared to free virus infection. (A)** Analysis of ZA110 wt and V1V2-deleted virus in T-cell to T-cell transmission using Jurkat donor cells (expressing replication competent TN6^inGLuc^ reporter viruses) and A301-CCR5 target cells (orange). Cell-cell transmission activity was compared to free virus infectivity of the same Jurkat derived viruses on TZM-bl cells (black). Data are normalized to ZA110 wt. The relative efficacy of cell-cell transmission versus free virus infection of the V1V2 mutant is shown below the bars. Mean and SD from 2 independent experiments are depicted. **(B)** A panel of 10 wt and V1V2 deletion mutant pseudoviruses (env-pv^inGLuc^) were compared in free virus infection of TZM-bl cells (black) and in cell-cell transmission from 293-T cells to A3.01-CCR5 cells (red). ΔV1V2 env mutant infectivity is normalized to each corresponding wt env. Values of relative efficacy of cell-cell transmission versus free virus infection are shown below the bars; a star indicates whether this difference is statistically significant as probed by multiple unpaired t-tests with alpha = 0.05. Mean and SD from 3 independent experiments are depicted. **(C)** The same env panel as shown in **(B)** was tested for free virus infection (black) and cell-cell fusion (green). Strain ZM109 ΔV1V2 was not infectious as free virus on TZM-bl cells (marked by a cross). ΔV1V2 env mutant infectivity or fusion activity were normalized to each corresponding wt env. Values of relative efficacy of cell-cell fusion versus free virus infection are shown below the bars; a star indicates whether this difference is statistically significant as probed by multiple unpaired t-tests with alpha = 0.05. Mean and SD from 3 independent experiments performed in duplicates are depicted.

Since the JR-FL V2 mutants L175P and D180N showed strong reduction in both free virus and cell-cell transmission infectivity we were interested to define the overall impact of the V1V2 domain on virus transmission. To this end we probed a panel of 10 viruses, encompassing subtype B Tier-1A viruses (SF162, NL4-3), subtype B Tier-2 viruses (JR-FL, RHPA, AC10, REJO, ZA110) and subtype C Tier-2 viruses (CAP88, ZM109, ZM214) to support free virus infection, cell-cell transmission and cell-cell fusion as wt and upon V1V2 deletion (Figure 3). While V1V2 deletion will not occur *in vivo*, the deletion mutants allowed us to obtain an assessment of the general contribution of the V1V2 domain to the different entry pathways across different virus strains as the impact of single point mutations can differ based on the envelope background probed.

When we assessed the infectivity of strain ZA110 in presence and absence of V1V2 in T-cell to T-cell transmission using Jurkat donor and A3.01-CCR5 target cells, we found that free virus infectivity of the V1V2-deleted env was reduced to 32% of wt level (Figure 3A). In contrast, cell-cell transmission capacity was maintained at 65% of wt level, representing a 2-fold higher efficacy of cell-cell transmission capacity compared to free virus infection (Figure 3A).

We extended our analyses to the panel of 10 wt and V1V2-deleted envs and probed them in 293-T to A3.01-CCR5 cell-cell transmission (Figure 3B). In agreement with previous reports [3,9,19-25], free virus entry capacity was strongly reduced upon V1V2 deletion. As observed for JR-FL V2 point mutants (Figure 1 and 2), the capacity of V1V2-deleted envs to support cell-cell transmission was lower compared to wt envs but generally exceeded free virus infectivity (Figure 3B). One exception was NL4-3 ΔV1V2, the only virus in our panel which proved not to excel free virus infectivity during cell-cell transmission. The other exceptions were SF162 ΔV1V2 and ZM214 ΔV1V2, which both retained cell-cell transmission activity at or close to wt level (96% and 83% of wt, respectively). This was especially notable for ZM214 as this virus, in contrast to SF162, showed markedly decreased infectivity as free virus upon V1V2 deletion. For the Tier-1A env clone SF162 the V1V2 domain seems to be completely dispensable for both free virus entry and cell-cell transmission. This is in line with the presumed open trimer conformation of SF162 in which the V1V2 does not perform the extent of inter-protomeric contacts required for shielding [9,20]. In sum, with the exception of NL4-3, all V1V2-deleted viruses retained partial cell-cell transmission activity, with no or comparatively lower loss in activity as seen during free virus transmission. The observed relative activities were 1.5 to > 8-fold higher in cell–cell transmission than in free virus infection (Figure 3B). Since both free virus infection and cell-cell transmission of V1V2-deleted envs were strongly reduced compared to wt, we ascertained that the RLU signals obtained in the respective entry assays were clearly above background and within the dynamic range of the assays (Additional file 3).

Interestingly, the capacity of V1V2-deleted envs to mediate cell-cell fusion remained in most cases intact (Figure 3C; 40 to 120% of wt fusion activity). Fusogenicity and free virus infectivity were not tightly linked in our panel, as for instance AC10, an envelope with the lowest free virus infectivity upon V1V2 deletion, retained fusion activity at almost wild type levels (Additional file 4A). In contrast, the other three viruses with comparatively low entry efficacy upon V1V2 deletion (JR-FL, RHPA and ZM109) all showed reduced fusion capacity (60.5%, 58% and 51.5% of wt fusogenicity, respectively). Overall, cell-cell fusion activity was maintained at higher levels (up to >25-fold relative activity over free virus infectivity) than cell-cell transmission (Figure 3B and C).

### Influences of the V1V2 domain on virion trimer content and stability

**Figure 4:**
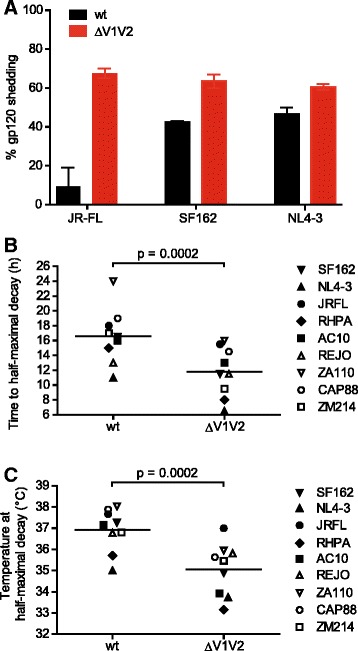
**V1V2 deletion impacts on trimer stability. (A)** Susceptibility of wt (black) and ΔV1V2 envs (red) to CD4-induced shedding was probed on env-transfected 293-T cells by flow cytometry. Percent of gp120 shedding upon incubation of env-expressing cells with CD4-IgG2 in relation to mock-treated controls is shown. Bars depict mean and SD of 2 independent experiments. **(B)** The half-life of wt and ΔV1V2 env virus stocks was determined by incubation for up to 80 h with periodic sampling and infectivity assessment on TZM-bl cells. Data points are means from 2 to 3 independent experiments. **(C)** Wt and ΔV1V2 virus stocks were subjected for 5 h to temperature gradient from 25°C to 45°C in 2.5°C steps followed by infectivity assessment on TZM-bl cells. The temperatures at which 50% infectivity of each stock remained are depicted. Data points are means of 2 to 3 independent experiments performed in duplicates.

While V1V2-deleted envs were expected to show pronounced defects in free virus entry [3,9,19-25], the high capacity of these envs to retain cell-cell transmission and cell-cell fusion activities was surprising. We therefore sought to dissect functions of V1V2 that are required for free virus entry but are, at least partially, dispensable during cell-cell transmission. Since V1V2 is a central component of the trimer required to maintain its integrity and stability [3,9,19-25], V1V2 deletion could potentially inflict a lower density of functional env trimers on virions that may cause the observed loss of free virus infectivity. To investigate potential decreases in env content we probed whether V1V2 deletion results in lower env expression on producer cells, decreased env incorporation into virions, or an increased propensity to CD4 induced gp120 shedding from trimers (Figure 4 and Additional file 5). Expression of V1V2-deleted envs on transfected 293-T cells was comparable to the corresponding wt envs (80 to >100% of wt; Additional file 5A and B). Similarly, levels of ΔV1V2 gp120 content of virions was in the range of wt for 8 of the 10 viruses probed (>80% of wt content, Additional file 5C). The strongest decrease was observed for ZM109 ΔV1V2, which only reached 10% of wt ZM109 gp120 levels and was not functional in free virus entry (Figure 3C). The second strain with lower gp120 content on virions was JR-FL ΔV1V2 which reached only 40% of wt levels. A more marked difference between wt and ΔV1V2 envs was apparent in their susceptibility to CD4-induced gp120 shedding, with V1V2-deleted envs showing a higher propensity to gp120 dissociation upon CD4 binding in line with previous reports on the reduced stability of V1V2 deleted envs [26,27] (Figure 4A). To probe the physical stability of ΔV1V2 virions in more detail, we analyzed temperature-induced virus infectivity decay in two different assays. The half-life of wt and V1V2-deleted virus stocks was determined by incubating virus samples at 37°C for up to 80 h and determining infectivity in regular intervals (Figure 4B and Additional file 6). The stability of wt envs proved to vary over a wide range. The most rapid decay was observed for the Tier-1A strain NL4-3, the highest stability displayed the Tier-2 env ZA110 with an half maximal decay time of 24 h. Interestingly, V1V2-deleted viruses displayed significantly faster decay rates than wt envs (Figure 4B; paired *t* test p = 0.0002, mean time to half maximal decay 16.6 h for wt and 11.8 h for ΔV1V2 envs). We further performed a temperature escalation treatment of wt and ΔV1V2 viruses by exposing virus aliquots to a temperature gradient ranging from 25° to 45°C. When we compared the temperatures at which virus stocks had lost 50% of their infectivity, we observed a markedly higher sensitivity of V1V2-deleted envs to increasing temperatures (Figure 4C and Additional file 7; paired *t*-test p = 0.0002; mean temperature at which 50% infectivity loss occurred 36.9°C and 35.1°C for wt and ΔV1V2 envs, respectively). Infectivity decay rates (Figure 4B) and temperature sensitivity (Figure 4C) of wt and ΔV1V2 envs correlated (wt envs: r = 0.825, p = 0.0062; ΔV1V2 envs: r = 0.734, p = 0.0243; Additional file 8). In summary, although we observed a range of reactivities for both wt and ΔV1V2 envs in terms of trimer content and stability, none of the probed parameters by itself proved to shape free virus and cell-cell infectivity (Additional file 4B-G). For instance, although V1V2-deleted JR-FL, AC10 and RHPA had a comparably low infectivity as free virus, only JR-FL proved to incorporate lower envelope densities (Additional file 5C). Despite this, the JR-FL ΔV1V2 env was among those envs which were closest to their respective wt in terms of decay rates and temperature stability (Figure 4B and C).

### The V1V2 domain is required for shielding against antibody neutralization during cell-cell transmission

A key function of V1V2 is shielding of the envelope trimer against neutralizing antibodies [3-13]. However, whether shielding by V1V2 is critical for both free virus infection and cell-cell transmission is currently not known as previous analyses have focused solely on free virus infection. Considering that efficacy of neutralizing antibodies is substantially reduced during cell-cell transmission [49,58,59,64], we thought it prudent to probe whether V1V2 shielding may be of less importance in this entry mode. To assess antibody neutralization during cell-cell transmission we utilized 293-T donor and A3.01-CCR5 target cells, for free virus neutralization 293-T produced virus stocks and TZM-bl target cells and for fusion inhibition 293-T donor and TZM-bl target cells. As shown above (Figure 1), utilizing TZM-bl target cells for free virus neutralization had the advantage that also virus stocks with low infectivity that showed only marginal infectivity of A3.01-CCR5 cells could be probed. Using JR-FL and a panel of neutralizing Abs we verified that free virus neutralization yielded identical results on A3.01-CCR5 and TZM-bl cells (data not shown).

**Figure 5:**
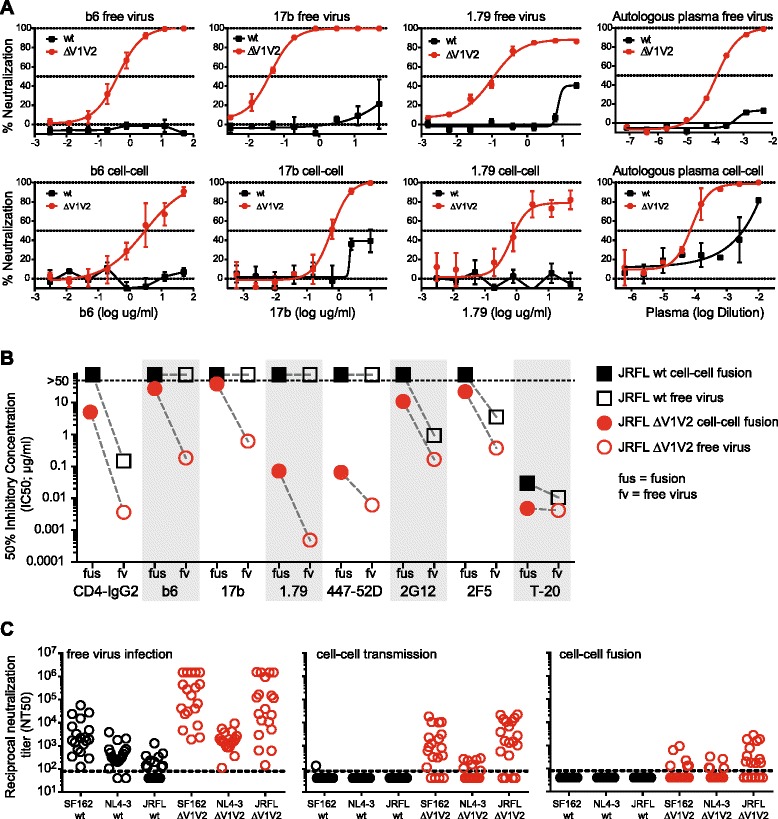
**V1V2 neutralization shielding during cell-cell transmission. (A)** to **(C)**: Neutralization activity of mAbs and patient plasma was evaluated in i) free virus infection by infection of env-pv^NLLuc^ viruses on TZM-bl cells; ii) during cell-cell fusion by co-culture of 293-T cells expressing env-pv^NLGFP^ and TZM-bl target cells; and iii) during cell-cell transmission by co-culture of 293-T cells expressing env-pv^inGLuc^ and A3.01-CCR5 target cells. **(A)** Neutralization sensitivity of isolate ZA110 wt (black squares) and ZA110 ΔV1V2 (red circles) to monoclonal antibodies b6, 17b and 1.79 and to autologous patient plasma was probed during free virus infection (top row) and cell-cell transmission (bottom row). Data points depict % neutralization compared to virus infection in absence of inhibitors. Mean and SD from 2 independent experiments performed in duplicate are shown. **(B)** Inhibition of JR-FL wt (black squares) and JR-FL ΔV1V2 (red circles) in free virus infection (open symbols) and cell-cell fusion (filled symbols) by mAbs and the fusion inhibitor T-20. Fifty percent inhibitory concentrations (IC50) in μg/ml calculated from pooled inhibition data derived from 2 to 3 independent experiments performed in duplicate are shown. **(C)** Neutralization activity of plasma from 19 individuals with chronic HIV-1 infection (subtypes A, B, C, AE, AG) against wt and ΔV1V2 mutant viruses of JR-FL, SF162 and NL4-3 was probed in free virus infection (left panel), cell-cell transmission (middle panel) and cell-cell fusion (right panel). The 50% neutralization titer (NT50, i.e. the reciprocal plasma dilution yielding 50% neutralization) is depicted. Neutralization titers were derived from 1 to 2 independent experiments, performed in duplicates.

We first investigated sensitivity of strain ZA110 as wt and upon V1V2 deletion against autologous plasma, the non-neutralizing CD4bs Ab b6, the moderately neutralizing V3-directed Ab 1.79 and the weak neutralizing CD4i Ab 17b during free virus infection and cell-cell transmission (Figure 5A). The Tier-2 ZA110 wt virus was not neutralized by any of the mAbs and only partially by the autologous plasma, irrespective if probed as free or cell-cell transmitted virus. V1V2 deletion strongly increased sensitivity of free virus to antibody neutralization. Sensitivity to neutralization during cell-cell transmission was also heightened upon V1V2 deletion, but the magnitude of the effect was lower. Cell–cell transmission of ZA110 ΔV1V2 required 6 to 14-fold higher antibody concentrations to reach 50% inhibition than free virus infection (50% inhibitory concentration (IC50) free virus infection vs. IC50 cell-cell transmission: b6 0.42 μg/ml vs. 2.97 μg/ml; 17b 0.041 μg/ml vs. 0.59 μg/ml; 1.79 0.1 μg/ml vs. 0.67 μg/ml).

When we compared V1V2 shielding of JR-FL during free virus infection and cell-cell fusion, we observed, in line with earlier reports [3-13], that free virus inhibition by V3, CD4bs and CD4i directed Abs was increased in absence of V1V2 shielding (Figure 5B). In contrast, gp41 directed agents (2F5 and T-20) and the gp120 glycan-specific Ab 2G12 showed no or only modest changes in activity against wt and V1V2-deleted viruses. As expected, cell-cell fusion of JR-FL wt proved resistant to antibodies while the activity of T-20 was retained. In agreement with free virus neutralization, V3 Abs showed highly improved activity in blocking cell-cell fusion in absence of V1V2. Surprisingly, this was not the case for CD4bs Ab b6 and CD4i Ab 17b, for which V1V2 deletion led to only small increases in neutralization sensitivity. This suggests that under conditions of rapid receptor engagement these weak or non-neutralizing Abs remain less active irrespective of V1V2 shielding.

We next compared plasma antibody activity of 19 individuals chronically infected with HIV-1 of subtypes A, B, C, AE and AG in blocking free virus infection, cell-cell transmission and cell fusion of strains SF162, NL4-3 and JR-FL wt and ΔV1V2 (Figure 5C). Free virus inhibition showed the previously described pattern, with plasma samples having considerable activity against the Tier-1A viruses SF162 and NL4-3 but only low activity against the Tier-2 virus JR-FL (SF162: inhibited by 19 samples at reciprocal 50% neutralization titers (NT50) up to 10^5^; NL4-3: inhibited by 17 samples at NT50 up to 10^4^; JR-FL: inhibited by 10 samples at NT50 up to 10^3^). V1V2 deletion increased neutralization activity against SF162 and JR-FL free virus (NT50 up to 10^6^) in line with improved exposure of V3, CD4bs and CD4i epitopes. Increase in neutralization sensitivity of NL4-3 upon loss of V1V2 was limited as plasma antibodies to V3 only rarely cross react with the V3 loop of this X4 user [13]. In contrast to free virus inhibition, none of the 19 plasma samples was capable of blocking cell-cell fusion of wt virus and only a fraction of the samples had activity against V1V2-deleted viruses, highlighting that cell-cell fusion is less prone to antibody attack even in absence of V1V2 shielding (SF162 ΔV1V2: inhibited by 6 samples; NL4-3 ΔV1V2: inhibited by 5 samples; JR-FL ΔV1V2: inhibited by 11 samples, all at NT50 below 10^4^). While wt viruses were equally insensitive to plasma inhibition during cell-cell transmission, cell-cell transmitted viruses lacking V1V2 portrayed an intermediate sensitivity with plasma neutralization potency ranging between free virus neutralization and cell fusion neutralization titers (SF162 ΔV1V2: inhibited by 15 samples at NT50 below 10^5^; NL4-3 ΔV1V2: inhibited by 7 samples at NT50 below 10^3^; JR-FL ΔV1V2: inhibited by 15 samples at NT50 below 10^5^). In summary, these data confirm that in cell-cell transmission, similar to free virus infection, intact V1V2 shielding is important for the virus to evade plasma antibody neutralization. However, the consequences upon loss of shielding by V1V2 are less pronounced during cell-cell transmission where neutralization titers were up to 3 orders of magnitude lower than in free virus transmission. Hence, imperfectly shielded viruses are highly likely to benefit from replicating in a cell-cell transmission environment.

### Selected V1V2 point mutations differentially affect free-virus infection and cell-cell transmissibility

**Figure 6:**
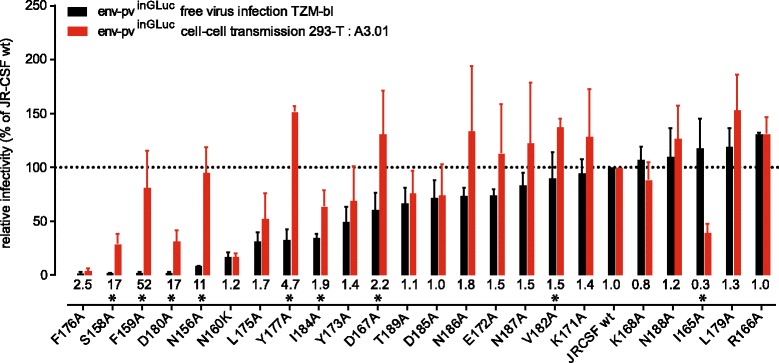
**Point mutations in V1V2 reduce free virus infectivity stronger than cell-cell transmission capacity.** A panel of JR-CSF V1V2 point mutations was compared for entry efficacy in free virus infection (black) and cell-cell transmission (red). Env mutant infectivities were normalized to JR-CSF wt and are ranked (left to right) in order of increasing free virus infectivity. Values of relative efficacy of each mutant in cell-cell transmission over free virus infection are indicated below the bars; stars depict whether this difference reached statistical significance as probed by multiple unpaired t-tests with alpha = 0.05. Data shown are means and SD from 3 independent experiments performed in duplicates.

While V1V2 deletion mutants served as important tool to dissect the role of V1V2 in the different entry pathways, we thought it prudent to verify the potential *in vivo* relevance of the observed effects in the context of naturally occurring V1V2 mutations. To investigate which positions in V1V2 are critical to preserve free virus infectivity we compared free virus infection, cell-cell transmission and cell-cell fusion capacity of a panel of 24 JR-CSF envs containing mutations of selected residues in V1V2 to alanine [65]. The panel includes residues forming epitopes of previously described V1V2-dependent antibodies [66,67] and/or being part of potential N-linked glycosylation sites shown to be critical for virus infectivity. When we tested the panel in free virus infection and cell-cell transmission we observed strong decreases in free virus infectivity for several of the mutants (Figure 6). Although cell-cell transmission capacity of several mutants was also reduced, infectivity was maintained at higher levels than in free virus infection. The difference was most pronounced for mutants that had the highest impact on free virus infection, including residues 156, 158, 159, 160, 177 and 180. Eight mutants retained free virus infectivity close to wt level (>90%) or even excelled it. While in all these cases cell-cell transmission was equally high, the I165A mutant was unique, as it was the only mutant that lost cell-cell transmission activity while maintaining high free virus infectivity. Although cell-cell transmission lead to a lower loss in infectivity across all mutants, free virus infectivity and cell-cell transmission capacity were correlated (r = 0.57, p = 0.0036, Additional file 9A) indicating that functional properties of the envs exist that govern both transmission modes. This functional link between free virus and cell-cell transmission for the majority of envs was even more evident when envs with high cell-cell transmission capacity and low free virus infectivity (N156A, F159A and Y177A) and the I165A mutant (showing the reverse phenotype), were excluded prior to correlation analysis (r = 0.87, p < 0.0001, Additional file 9B). The cell-cell fusion capacity of the env panel showed a similar pattern with fusion capacity being maintained at much higher rates than free virus infectivity. Fourteen viruses reached ≥ 75% of wt fusion levels and only 4 viruses showed fusion activities below 50% of wt fusion capacity (Additional file 9C).

**Figure 7:**
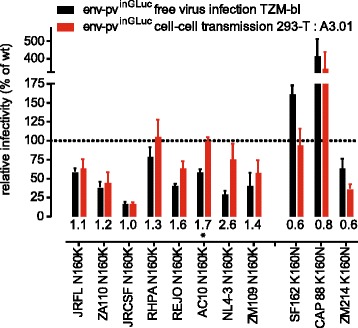
**The effect of residue N160 on free virus infection and cell-cell transmission.** Eight viruses encoding N160 and 3 viruses encoding K160 were probed as wt and upon N160K or K160N mutation in free virus infection (black) and cell-cell transmission (red). Mutant env infectivity in the two transmission pathways is normalized to the matching wt env. Values of relative efficacy of each mutant in cell-cell transmission over free virus infection are indicated below the bars; stars depict whether this difference reached statistical significance as probed by multiple unpaired t-tests with alpha = 0.05. Data shown are means and SD from 2 to 3 independent experiments performed in duplicates.

In a next step we focused our analyses on V1V2 residue 160 which is a crucial component of the epitope of a class of V1V2-directed broadly neutralizing antibodies represented by PG9 and PG16 [31]. Escape from these antibodies is associated with loss of asparagine at position 160 (N160) which is conserved in more than 90% of HIV-1 sequences deposited in the Los Alamos HIV sequence database (3719 env sequences included; [68]). PG9/PG16 resistant strains frequently carry a lysine at position 160 (K160). K160 is found in 2.4% of env sequences in the Los Alamos database. Among viruses probed in our study, 3 envs (SF162, CAP88 and ZM214) encode lysine at position 160. To test the effect on the entry phenotype of this residue, we generated a panel of mutant N160K or K160N viruses and analyzed their free virus infection and cell-cell transmission capacity. In agreement with previous studies [69], the effect of N160K on free virus entry ranged from a 10 to 90% loss of entry capacity for the different isolates tested (Figure 7). Notably, in two of the three strains which naturally contain a lysine at position 160, SF162 and CAP88, the K160N mutation reconstituted a potential N-linked glycosylation site and led to a high increase in free virus infectivity, highlighting the importance of this residue for free virus infectivity. In contrast, for strain ZM214 we did not observe a boost in infectivity upon introduction of K160N. Interestingly though, in ZM214 the K160N mutation does not restore an N-x-S/T motif, suggesting that glycosylation at position 160 is required for the observed boost in free virus infectivity. When we tested the panel of N160K mutants in cell-cell transmission (Figure 7) the overall picture was more diverse, indicating that presence or absence of the glycosylation site at position 160 has strain-dependent effects on free virus infectivity and cell-cell transmission capacity. Out of the 8 probed mutants, 3 envs had identical or only moderately increased (<10% difference) activity in cell-cell transmission compared to free virus transmission. The remaining 5 viruses showed a higher efficacy of cell-cell transmission (ranging from a 15 to 40% increase in efficacy compared to free virus infection). SF162 and CAP88 K160N mutant cell-cell transmission efficacy was either equal to wt (SF162) or in the range of free virus transmission (CAP88), but did not excel it, suggesting that glycosylation at position 160 has a greater effect on free virus infectivity than on cell-cell transmission. Cell-cell fusion capacity of the mutants was in all cases maintained at high levels (65 to 100% of wt; Additional file 10).

### Infectivity differences between SF162 and P3N are steered by V1V2 and N160

**Figure 8:**
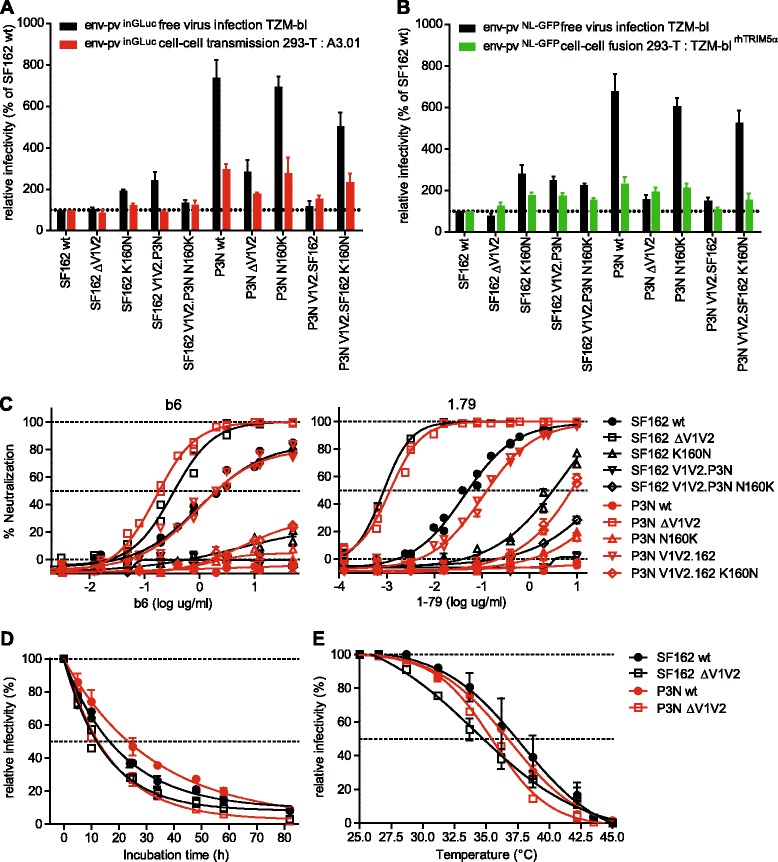
**V1V2 function analysis of strains SF162 and P3N. (A)** Activity of SF162 and P3N wt and mutant envs was compared in free virus infection of TZM-bl cells (black) and cell-cell transmission from 293-T cells to A3.01-CCR5 cells (red). Data are normalized to SF162 wt. **(B)** Comparison of the same virus panel shown in **(A)** in free virus infection of TZM-bl cells (black) and cell-cell fusion activity between 293-T cells and TZM-bl^rhTRIM5α^ cells (green). Data are normalized to SF162 wt. **(C)** Free virus neutralization of env-pv^NLLuc^ pseudoviruses by anti-V3 mAb 1.79 (right) and anti-CD4bs mAb b6 (left). **(D)** Assessment of virus temperature stabilities upon long-term incubation of P3N and SF162 wt and ΔV1V2 virus stocks. **(E)** Assessment of virus temperature stabilities upon temperature gradient incubation of P3N and SF162 wt and ΔV1V2 virus stocks. **(A)** to **(E)**: All data shown are mean and SD from 2 to 3 independent experiments performed in duplicates.

The differences in entry efficacy we observed for V1V2 point mutants, in particular N160 variants, were intriguing. To obtain further insights into the interplay of V1V2 variability and infectivity we utilized the Tier-1A strain SF162 and its Tier-3 derivative P3N, which was derived from *in vivo* evolution via successive transfer of virus in rhesus macaques following initial infection with SHIV-SF162 [70]. Of particular interest for our study, strain P3N has substantial amino acid changes compared to SF162 in V1V2 (Additional file 11), including a switch from the lysine found at position 160 in SF162 to asparagine restoring an N-linked glycosylation motif. We therefore analyzed the effect of switching between lysine and asparagine in the parental SF162 and P3N envelopes. In addition we generated a V1V2-deleted version of P3N, as well as swaps of the V1V2 loops between SF162 and P3N. The panel of wt SF162 and P3N and their derivatives containing domain swaps and/or mutations of position 160 were analyzed for free virus infectivity, cell-cell transmission and cell-cell fusion activity (Figure 8A and B). P3N is highly infectious compared to SF162; however when V1V2 was deleted or replaced with the V1V2 of SF162, the infectivity of P3N dropped to a similar level as SF162, highlighting the importance of V1V2 for the high infectivity of P3N. The opposite effect was less pronounced, as the V1V2 of P3N inserted into SF162 lead to only an approximately 2-fold increase in free virus infectivity, indicating that additional mutations outside V1V2 are required to restore the full infection potential seen for P3N. The N160K mutation in P3N had a negligible impact on infectivity, suggesting that inter-protomeric contacts of P3N are highly optimized and do not depend on a single interaction by N160. In striking contrast, the infectivity of P3N carrying the V1V2 of SF162 could be restored by introduction of N160, highlighting the importance of the N160 glycan for protomer interactions of certain V1V2 domains. When we tested the env panel for cell-cell transmission we observed a similar trend, with SF162-derived mutants remaining close to SF162 wt levels. Notably, P3N wt was approximately 3-fold better than SF162 wt in cell-cell transmission, but more than 7-fold better in free virus infection (Figure 8A). This further underscores that envs less fit in free virus infection benefit more from cell-cell transmission, while the advantage of free virus entry competent envs is less pronounced in this entry pathway. Similar to free virus infection, the loss in cell-cell transmission capacity observed for the P3N chimera carrying the SF162 V1V2 could be restored by introduction of K160N confirming the importance of this residue in both entry modes. Cell-cell fusion capacity of all envs ranged from 100% to 250% of SF162 wt activity (Figure 8B).

SF162 and SF162 ΔV1V2 are highly neutralization sensitive viruses (Tier-1A), whereas P3N is highly neutralization resistant (Tier-3). When V1V2 was deleted from P3N, it showed a neutralization phenotype as SF162 ΔV1V2 and was potently neutralized by the V3 specific Ab 1.79 and the CD4bs Ab b6 (Figure 8C). Similarly, replacement of the P3N V1V2 with that of SF162 lead to a neutralization-sensitive phenotype comparable to SF162 wt. In contrast, the V1V2 of P3N introduced neutralization resistance comparable to P3N wt in SF162. Of note, the K160N mutation in both SF162 and P3N.V1V2SF162 resulted in a high increase in neutralization resistance. Thus V1V2, and especially the residue at position 160, is important for maintaining a closed, neutralization-resistant and entry-competent trimer conformation. Probing the association between sensitivity to mAbs 1.79 and b6 and free virus infectivity of the eight viruses confirmed this relationship (correlation analysis: b6 r = 0.809, p = 0.015; 1.79 r = 0.775, p = 0.0238; Additional file 12). To further determine the influence of V1V2 on entry characteristics, we analyzed the SF162 and P3N wt and V1V2-deleted envs for trimer stability. Analysis of temperature stability indicated that P3N was the most stable env out of the four envs tested (Figure 8D and E), providing further evidence that trimer stability is tightly linked with entry competence and neutralization resistance.

## Discussion

The V1V2 domain of gp120 is a key regulator of virus infectivity and shielding against neutralizing antibody attack [3-13,19-25]. Rapid antibody escape mediated by mutations in V1V2 highlights the latter activity and is considered the main driver of the high sequence diversity of the domain [14,15]. How the domain supports this high plasticity without jeopardizing entry functionality of the envelope trimer, for which it contributes essential, stabilizing intra- and inter-protomeric contacts [17,18] has not been conclusively resolved. We hypothesized that preserving entry capacity in the face of continuously evolving neutralization escape mutations will require means for the virus to quickly adopt compensatory mutations which rescue infectivity. A prerequisite for this would be that the escape mutants that arise maintain residual entry capacity. We reasoned that cell-cell transmission, being a more efficient mode of virus dissemination than free virus infection [49-57], may play an important role in this context. Since effects of V1V2 variability on cell-cell transmission efficacy have not been assessed to date, we set out in this study to systematically delineate factors that shape entry efficacy of V1V2 variants during free virus infection and cell-cell transmission.

When we tested a panel of 10 HIV-1 envs as wt and upon V1V2 deletion, we observed a strong reduction of free virus entry efficiency of V1V2 deleted viruses while their cell-cell transmission and cell-cell fusion capacity was maintained at considerably higher levels (Figure 3). To investigate V1V2 functions that shape entry efficacy we analyzed virion gp120 content, trimer stability and neutralization phenotype (Figures 4 and 5). Expression and virion incorporation levels of wt and V1V2-deleted envs were, with few exceptions, in a comparable range (Additional file 5). However, upon incubation with soluble CD4, the propensity of V1V2 deleted env to gp120 shedding, and therefore trimer inactivation, was increased. Additionally, we observed faster rates of infectivity decay upon prolonged incubation at 37°C and a decreased tolerance to temperature increases of V1V2-deleted viruses compared to wt. Both observations, higher rates of CD4-induced shedding and faster temperature-induced infectivity loss of V1V2-deleted envs compared to wt, are in agreement with previous studies showing high propensities of V1V2-mutant env to negotiate transitions to low-energy states and trimer inactivation [26,27]. We further found that the V1V2 domain is required for shielding of the trimer against neutralizing antibodies during cell-cell transmission. However, increases in antibody potency upon V1V2 deletion were less pronounced than in free virus infection, indicating that viruses with defects in V1V2 that decrease shielding capacity may benefit from replicating by cell-cell transmission.

A particular emphasis of our study was on studying the influence of physiologically relevant V1V2 mutations on the two entry pathways. We probed a comprehensive panel of V1V2 point mutations and observed similar patterns as with V1V2 deletion, namely an increased capacity of the mutants to retain infectivity in cell-cell transmission while free virus infection was, in several cases, severely impaired. Of particular interest, we observed two “hot spots” where mutations caused strong reductions in free virus entry capacity while cell-cell transmission was retained at significantly higher levels, suggesting that these regions are particularly important for ensuring trimer functionality. These regions were located between residues 156 to 160 and 175 to 180 (Figure 6). The region encompassing residues 156–160 is part of an epitope targeted by antibodies [31-33,48,71], including the broad and potent antibodies PG9, PG16 and PGT145. It is tempting to speculate that this region is preferentially targeted by Abs due to more stringent structural or sequence constraints compared to the rest of V1V2. We explored the entry phenotype of V2 position 160 in more detail, as this residue is crucial for the formation of the epitope of PG9-like antibodies [31,33,34]. Asparagine, as part of an N-linked glycosylation sequon, is the predominant residue at this position with over 90% sequence conservation [68]. We found that switching between asparagine and lysine (N160K) can dramatically alter both env entry fitness during free virus infection and neutralization phenotype, in agreement with previous studies (Figures 7 and 8) [69,72]. Notably though, cell-cell transmission capacity was less affected than free virus infection by alterations at position 160, supporting the notion that neutralization escape variants requiring N160 mutation may be rescued via cell-cell transmission. Re-introduction of the glycosylation site at position 160 in strains SF162 and CAP88, which naturally lack a glycosylation motif at this position, induced a strong increase in free virus infectivity. Considering the high conservation of this site across HIV-1 subtypes this may indicate the importance of this glycosylation for HIV to maintain free virus spread in natural infection.

The second hot spot we identified encompasses residues 175 to 180 which have been postulated to mediate gp120 binding to the α4β7 gut homing receptor. Several reports suggested that binding to α4β7 fosters efficient infection of T-cell subsets expressing this receptor [73,74], while other studies challenged this view [75,76], currently leaving the physiological importance of α4β7 interaction unresolved. When we analyzed envs containing mutations of residues that form part of the tripeptide motif LDI/V at the tip of V2 required for α4β7 binding, we found that especially mutation of the highly conserved aspartate at position 180 induced strong reductions in free virus infectivity, while cell-cell transmission capacity was maintained at higher levels. This phenotype was observed both for JR-FL (Figure 1 and 2) and JR-CSF (Figure 6). A3.01-CCR5 cells used in our study can express α4β7 [77], whereas TZM-bl cells do not. Thus, the strong effect we observed upon mutation of residue 180 may not necessarily reflect a lack of binding to this receptor, but alternatively may indicate the importance of this highly conserved residue for V2 conformation [78,79].

Recent years have substantiated that cell-cell transmission is an effective means of virus transmission, at least *in vitro,* due to a concentration of virus and receptor molecules in the donor-target cell contact area, rapid infection which outcompetes spontaneous virus inactivation, and a high multiplicity of target cell infection [49-57]. The relevance of cell-cell transmission *in vivo* remains unresolved [80]; however, cell-cell transmission and even cell-cell fusion were recently demonstrated to occur between T-cells in HIV-infected humanized mice [81]. Our observations that viruses carrying envelopes with defects in V1V2 perform better in cell-cell transmission than in free virus infection further highlight the potential for the virus in utilizing the cell-cell transmission pathway. Indeed, our findings may lend themselves towards an improved understanding of virus evolution and spread *in vivo*. V1V2 is notable for its high sequence diversity, both within the swarm of quasispecies of an infected host as wells as across different HIV subtypes, and may even adopt different conformations on the env trimer [67,82,83]. The high sequence variation and structural plasticity of V1V2 is likely required to escape antibody neutralization, including direct escape of V1V2-targeting antibodies and adaptation of its shielding capacity of distal epitopes such as the V3 loop [3-13]. Given the importance of V1V2 for trimer integrity and entry, the obvious question is: how can V1V2 functionality be maintained despite the constantly high sequence variation? Based on our observation that mutations in V1V2 that strongly decrease free virus infectivity can retain cell-cell transmission capacity, we propose that cell-cell transmission may constitute a salvage pathway for the virus (Additional file 13). Upon antibody escape mutations in V1V2 that decrease free virus infectivity, the virus may retract to cell-cell transmission until compensatory mutations, either within or outside V1V2 [84-86], may emerge that restore the full infection potential. We suggest that this alternative virus escape pathway should be considered in future approaches towards HIV treatment and vaccine design.

## Conclusions

The V1V2 domain of the HIV-1 envelope glycoprotein gp120 combines two essential functions: It is a key regulator of virus infectivity ensuring the functionality of the envelope trimer and shields the trimer against neutralizing antibody attack. Rapid antibody escape induced by mutations in V1V2 highlights the latter activity and is considered the main driver of the high sequence variability of the domain. How the high mutation rate in V1V2 can be tolerated despite the need to maintain envelope trimer functionality is currently not completely understood. We hypothesized that preservation of entry capacity in the face of continuous neutralization escape mutations will require means for the virus to quickly adopt compensatory mutations which rescue infectivity. A prerequisite for the latter is that the initial escape mutants that arise maintain residual entry capacity. In this study we demonstrate that virus cell-cell transmission, due to its capacity to excel free virus infectivity, can indeed partially rescue infectivity of V1V2 mutant viruses. Hence, cell-cell transmission may provide an important salvage pathway for replication of HIV antibody escape variants until compensatory mutations emerge that restore free virus infection potential.

## Methods

### Antibodies and inhibitors

MAbs were kindly provided by: 2F5 [87] and 2G12 [88] by Dr. Dietmar Katinger, Polymun Scientific, Vienna, Austria. b6 [89] by Dr. Dennis Burton, The Scripps Research Institute, La Jolla, USA. 17b [90] by Dr. James Robinson, Tulane University, New Orleans, USA. 447-52D [91] was purchased from Polymun Scientific. Expression plasmids for antibody 1.79 [92] were provided by Dr. Michel Nussenzweig, The Rockefeller University, New York, USA. Antibody 1.79 was produced by expression in 293-F cells followed by purification of the antibody by protein G affinity chromatography and size exclusion chromatography as described [13]. T-20 [93] was purchased from Roche Pharmaceuticals.

### Cells and viruses

293-T cells were obtained from the American Type Culture Collection (ATCC) and TZM-bl cells [94] from the NIH AIDS Research and Reference Reagent Program (NIH ARP). Both cell types were cultivated in DMEM containing 10% heat inactivated FCS and penicillin/streptomycin. A3.01-CCR5 T cells were described previously [58] and were cultivated in RPMI containing 10% FCS and penicillin/streptomycin. Jurkat T cells [95] were a gift from Dr. Stuart Neil, King’s College, London, UK and were cultured in RPMI, 10% FCS and penicillin/streptomycin. rhTRIM5α-expressing TZM-bl cells were described previously [58].

All envelope genes used in this study were cloned into the expression vector pcDNA 3.1 (Invitrogen). Plasmids encoding the envelopes of strains JR-FL, SF162, NL-43, RHPA, AC10, REJO, ZM109 and ZM214 were obtained from the NIH ARP. Envelope clone SF162-P3N [70] was a gift from Dr. Cecilia Cheng-Mayer, Aaron Diamond AIDS Research Center, New York, USA. Envelope ZA110 was previously described [13]. Envelope clone CAP88 [96] was a gift from Dr. Lynn Morris, National Institute for Communicable Diseases, Johannesburg, South Africa. The panel of JR-CSF alanine point mutants was kindly provided Dr. Dennis Burton [65]. For the construction of V1V2 deleted viruses the restriction sites DraIII (upstream) and StuI (downstream) of the V1V2 were utilized to substitute the original V1V2 domain with a three amino acid linker sequence DAG as described [13,97]. The same strategy was employed to produce V1V2 swaps between envelopes SF162 and P3N. All other mutations were generated by site directed mutagenesis (QuikChange II kit, Agilent, Santa Clara, USA) according to the manufacturer instructions.

### Clinical specimen

Plasma samples used for neutralization studies were obtained from 19 adult individuals with chronic HIV-1 infection (subtypes A, B, C, AE and AG, > 6 months infected) enrolled in the Zurich center of the Swiss HIV Cohort Study [98] and the Zurich Primary HIV-infection (ZPHI) study [99]. The plasma sample from patient ZA110 used in Figure 5, a participant of the ZPHI study, was collected 61 weeks before the time point at which the probed env was isolated [13].

### Ethics statement

All clinical specimens were derived from adult participants enrolled in the Zurich center of the Swiss HIV Cohort Study [98] or the ZPHI study [99]. All studies were approved by the ethics committee of the University Hospital Zurich and written informed consent was obtained from all individuals.

### Sequencing

All envelope sequence data are recorded based on HXB2 numbering. Sequencing of envelope genes was performed using in-house Sanger sequencing.

### Preparation of HIV-1 virus stocks

To prepare pseudovirus stocks 293-T cells were transfected with the respective viral backbones and the envelope of choice as described [100]. 48 h post-transfection, virus containing supernatants were harvested and infectivity was determined on TZM-bl reporter cells as described [100]. The following virus constructs were used: The GFP reporter HIV pseudotyping vector pNLGFP-AM ([58]; denoted env-pv^NLGFP^) was co-transfected with env encoding plasmids to compare free virus infectivity and cell-fusion (Figures 2 and 3). The Luciferase reporter HIV pseudotyping vector pNLLuc-AM ([13]; denoted env-pv^NLLuc^) was co-transfected with env encoding plasmids to probe free virus neutralization activity (Figures 5 and 8) and free virus stability and gp120/p24 content (Figure 4). The lentiviral packaging vector pCMV-dR8.91 ([101]; gift from Dr. Didier Trono, EPFL Lausanne, Switzerland) was co-transfected with an env encoding plasmid and the inGLuc reporter construct pUCHR-inGLuc ([56,62], kindly provided by Dr. Walther Mothes and Dr. Gisela Heidecker, see section on cell-cell transmission below for details). The obtained pseudoviruses (encoded env-pv^inGLuc^) were used for comparison of free virus and cell-cell transmission (Figures 1, 2, 3, 5, 6, 7, 8). To monitor cell-cell transmission in a T-cell to T-cell setting, we generated replication competent (rc) viruses containing the envelopes of JR-FL and ZA110 with the indicated mutations in the TN6 NL vector, a replication-competent viral backbone engineered from strain NL4-3 (kindly provided by Dr. M. Dittmar, Queen Mary University of London, UK) as described [102]. These env-TN6 encoding plasmids were co-transfected with the inGLuc reporter plasmid on Jurkat T cells (encoded env-rc TN6^inGLuc^) and used in free virus and cell-cell transmission experiments (Figures 2 and 3). Infectivity of env-pv^NLLuc^ and env-pv^NLGFP^ viruses on TZM-bl cells was quantified by measuring firefly luciferase activity using firefly luciferase substrate (Promega, Madison Wisconsin, USA). Infectivity of env-pv^inGLuc^ and env-rc TN6^inGLuc^ on target cells (TZM-bl and A3.01-CCR5) was quantified by measuring Gaussia luciferase activity using the Renilla-Glo Luciferase Assay System (Promega AG, Madison, USA).

### Comparing HIV-1 free virus infectivity and cell-cell transmission capacity

We employed the recently described inGLuc reporter system [56,62] to assess HIV cell-cell transmission. This setup employs a reporter plasmid (pUCHR-inGLuc) containing Gaussia luciferase in reverse orientation, interrupted by an intron in forward orientation. Splicing of the intron in plasmid-transfected HIV donor cells, packaging of the spliced RNA into virions followed by infection, reverse transcription and integration in a target cell leads to Gaussia luciferase reporter gene expression. Hence, no signal is induced in plasmid-transfected cells or from cell fusion ([56,62], Figure 1 and data not shown). Virus infectivity in this system is sensitive to both protease and RT inhibitors as expected for cell-cell transmission ([103] and Figure 1). To assess cell-cell transmission in absence of free virus transmission we restricted free virus infectivity by omission of DEAE-dextran (diethylaminoethyl-dextran; Amersham Biosciences, Fairfield, Connecticut, USA) in the infection media [58].

To directly compare free virus infectivity and cell-cell transmission, 293-T cells (2 wells of a 12-well plate per env; 100.000 293-T cells per well seeded one day before transfection) were transfected with env expression plasmid, inGLuc plasmid and the lentiviral packaging vector pCMV-dR8.91 (0.2 μg, 1.2 μg and 0.6 μg per well, respectively) using polyethyleneimine (PEI) as transfection reagent. 6 h post-transfection, one well for each env was processed for free virus infectivity, while the other well was used to probe cell-cell transmission.

#### Free virus infectivity

To obtain virus supernatant for estimating free virus infectivity, the supernatant of a transfected 12 well was aspired 6 h post transfection and replaced with 1 ml fresh complete DMEM. 48 h post-transfection, the virus-containing supernatant was harvested, cleared by centrifugation (300 g, 3’) and stored at −80°C. Subsequently free virus infectivity was determined by adding serial dilutions of virus stocks, starting with a maximal input of 100 μl, to TZM-bl or A3.01-CCR5 cells in 96-well plates in medium containing 10 μg/ml of DEAE-dextran and measuring Gaussia luciferase activity 60 h post-infection.

#### Cell-cell transmission

To measure cell-cell transmission, the supernatant from transfected 293-T cells was aspired 6 h post transfection and the cells were resuspended in 1 ml fresh complete RPMI medium containing no DEAE (yielding approximately 15.000 cells per 100 μl). Subsequently, cells were re-seeded in 96-well plates (100 μl per well), and per well 50.000 A3.01-CCR5 cells were added in 100 μl RPMI medium. After 60 h incubation at 37°C Gaussia luciferase production was measured to determine target cell infection.

### Assessing HIV-1 T-cell to T-cell transmission

In order to probe virus transmission from T-cells to T-cells we seeded Jurkat T-cells in 12-well plates at 500.000 cells per well in complete RPMI medium. Cells were transfected with infectious molecular clones of HIV (TN6 NL vector encoding different env) and the inGLuc reporter plasmid (2 μg and 0.5 μg per well, respectively) using Jurkat TransIT (Mirus Bio LLC, Madison, USA) as transfection reagent. 6 h post-transfection, the transfection medium was removed and we either (i) added 1 ml fresh complete RPMI, or (ii) resuspended cells in 1 ml RPMI, transferred 100 μl to 96-well plates, and added 50.000 A3.01 cells in 100 μl fresh complete RPMI. From the wells containing only Jurkat cells, the cell-free supernatant was harvested 48 h after the medium change and the free virus titer was determined on TZM-bl cells by Gaussia luciferase read-out as described above. From the wells containing the co-culture of Jurkat and A3.01 cells, a Gaussia luciferase read-out was performed 60 h after starting the co-culture.

### Comparing HIV-1 free virus infectivity and cell-cell fusion capacity

Free virus infectivity and cell-cell fusion activity was probed using 293-T cells producing env pseudotyped reporter virus (env-pv^NLGFP^) on TZM-bl target cells. 293-T cells were seeded in 12-well plates 1 day before transfection (100.000 cells per well in 1 ml complete DMEM). For each env, two wells of 293-T cells were transfected with env-encoding plasmid and reporter vector pNLGFP-AM (0.5 μg plus 1.5 μg per well, respectively) using PEI as transfection reagent. 6 h post-transfection, one well for each env was processed for free virus infectivity, while the other well was used to probe cell-cell fusion.

#### Free virus infectivity

For free virus infectivity, the transfection supernatant was aspired 6 h post transfection and replaced with 1 ml fresh complete DMEM. 48 h post-transfection, the virus-containing supernatant was harvested, cleared by centrifugation (300 g, 3’) and TZM-bl cells were infected with serial dilutions of the virus supernatant in medium containing DEAE-dextran. 48 h post infection firefly luciferase activity in the TZM-bl cells was measured.

#### Cell-cell fusion

Envelope fusogenicity was assessed by Tat driven induction of firefly luciferase in TZM-bl^rh-TRIM5α^ targets cells upon co-culturing with 293-T cells transfected with env and the viral backbone plasmid pNLGFP-AM. In TZM-bl^rh-TRIM5α^ cells free virus infectivity is potently restricted through expression of rhTRIM5α [58,104]. Co-culturing of env pseudovirus-expressing 293-T cells with TZM-bl^rh-TRIM5α^ cells leads to a rapid induction of the LTR driven luciferase reporter in the TZM-bl cells due to Tat transfer from transfected donor 293-T cells during fusion. To measure cell-cell fusion, the supernatant of a 12-well containing pNL-GFP AM and env transfected 293-T cells was aspired after 6 h, and the cells were resuspended in 1 ml fresh DMEM (yielding approximately 15.000 cells per 100 μl). Cells were then distributed in 96-well plates (100 μl per well), and per well 15.000 TZM-bl^rh-TRIM5α^ cells in 100 μl complete DMEM were added. After 24 h incubation at 37°C production of firefly luciferase was measured.

### Assessment of gp120 shedding

To probe propensity of different envs to CD4 induced shedding of gp120 from trimers, 293-T cells were seeded in 12-well plates (100.000 cells per well in 1 ml complete DMEM). 24 h later, cells were transfected with env-encoding plasmids and a rev-encoding plasmid (1.6 μg plus 0.4 μg per well, respectively). 8 h post-transfection the medium was replaced, and 48 h post-transfection the medium was aspired and cells resuspended in FACS buffer (PBS, 2 mM EDTA, 2% FCS). The cells were transferred to a 96-well plate and treated with sCD4 (10 μg/ml in FACS buffer) or mock (FACS buffer) for 30’ at 37°C. Subsequently, the cells were washed twice with FACS buffer and envelope on the cell surface was detected with biotinylated mAb 2G12 (5 μg/ml in FACS buffer) and Streptavidin-APC (BioLegend, San Diego, USA; 1:400 in FACS buffer) followed by analysis of the cells on a CyAN ADP flow cytometer (Beckman Coulter, Brea, USA). Percent shedding was calculated as the mean fluorescence intensity (MFI) of the sCD4-treated cells divided by the MFI of the mock-treated cells.

### Quantification of gp120 and p24 content of pseudotype virus stocks

To determine gp120 and p24 content of virus preparations, env-pv^NLLuc^ viruses were produced in 293-T cells (T25 flasks; 750.000 cells in 5 ml medium, seeded 24 h pre-transfection). The medium was changed 12 h post-transfection and virus-containing supernatants harvested 48 h post-transfection. The supernatants were cleared by low speed centrifugation (300 g, 3’), then ultracentrifuged (SW28 rotor, 2 h, 28.000 rpm, 4°C), the supernatant removed and viral pellets resuspended in 0.3 ml cold PBS and stored at −80°C. Virion associated p24 and gp120 antigens were quantified by ELISA as previously described [13]. Briefly, virus preparations were dissolved in 1% Empigen (Fluka Analytical, Buchs, Switzerland) and dilutions of each sample probed for gp120 and p24. Gp120 was captured on anti-gp120 D7324 (Aalto Bioreagents, Dublin, Ireland) coated immunosorbent plates and detected with biotinylated CD4-IgG2 and Streptavidin-coupled Alkaline Phosphatase (GE Healthcare, Chalfont St Giles, UK). P24 was captured on anti-gp120 D7320 (Aalto Bioreagents, Dublin, Ireland) coated plated and detected using Alkaline Phosphatase-coupled antibody BC1071-AP (Aalto Bioreagents, Dublin, Ireland).

### Assessment of virus temperature decay

We employed two different experimental setups to probe temperature-induced virus infectivity loss. In the first assay, 250 μl aliquots of wt and V1V2-deleted env-pv^NLLuc^ virus stocks were incubated at 37°C for up to 80 h. At defined time points, the titer of an incubated sample was determined by titration on TZM-bL cells and set in relation to the titer of a freshly thawed aliquot to retrieve the relative loss in infectivity over time of incubation at 37°C. For the second assay, virus stocks were transferred to 96-well PCR plates (125 μl per well) and incubated for 5 h over a temperature gradient ranging from 25°C to 45°C in 2.5° steps in a Biometra T-gradient 96-well PCR block (Biometra GmbH, Göttingen, Germany). Following incubation, the samples were titrated on TZM-bl cells and the infectivity of each sample was normalized to the sample incubated at 25°C (100% infectivity), yielding relative infectivity curves as a function of incubation temperature.

### Neutralization of cell-free env-pseudotyped virus

The neutralization activity of mAbs and patient plasma against cell-free env-pv^NLLuc^ viruses was evaluated on TZM-bl cells as described [100]. Virus input was chosen to yield virus infectivity corresponding to a luciferase activity of 5000 to 20.000 RLU in absence of inhibitors. The antibody concentrations or reciprocal plasma titers causing 50% reduction in viral infectivity (inhibitory concentration IC_50_ or neutralization titer NT_50_) were calculated by fitting pooled data from two to three independent experiments to sigmoidal dose response curves (variable slope) using GraphPad Prism. If 50% inhibition was not achieved at the highest or lowest antibody or plasma concentration, a greater-than or less-than value was recorded.

### Neutralization assay during cell-cell fusion and cell-cell transmission of env-pseudotyped virus

To assess neutralization during cell-cell fusion, 293-T cells transfected with plasmids encoding env-pv^NLGFP^ were re-seeded 6 h post transfection at 10.000 cells in 100 μl DMEM medium per well of 96 well plates and serial dilutions of inhibitors (50 μl per well) were added to the cells. Following a 1 h incubation at 37°C, TZM-bl^rhTRIM5α^ target cells were added to the 293-T cell – inhibitor mix (10.000 TZM-bl^rhTRIM5α^ cells per well in 50 μl DMEM). 24 h post co-culture start, firefly luciferase activity was quantified as described. To assess neutralization during cell-cell transmission, we used the same assay strategy with the following adaptations. 293-T cells were transfected with plasmids encoding env-pv^inGLuc^ and later co-cultured with 50.000 A3.01-CCR5 target cells per well in 96-well plates. Gaussia luciferase activity in the culture supernatant was quantified 60 h after co-culture. Neutralization assay data from both setups were processed in GraphPad Prism as described above for free virus neutralization.

### Statistical analysis

All correlations were performed in GraphPad Prism according to Pearson. Analyses for statistical significance were performed with GraphPad Prism using either paired t-tests (Figure 4B and C, with the exact p-values indicated) or multiple unpaired t-tests (Figures 2, 3, 6, 7) with statistical significance defined by alpha = 0.05.

## Additional files

Additional file 1:
**Assay setups to study free virus infection and cell-cell transmission.** **(A)** Overview of the assay formats used to dissect free virus infection and cell-cell transmission. **(B)** Experimental assay scheme: To study and quantitatively compare HIV free virus infection and cell-cell transmission virus donor cells (either 293-T cells or Jurkat T cells) were transfected with HIV encoding plasmids and the inGLuc reporter plasmid. Transfection was performed in 12-well plates, with two wells per env. Six hours post-transfection, one well was processed for cell-cell transmission, and one well for free virus infection: (i) To assess cell-cell transmission, the donor cells were resuspended in 1 ml medium and re-seeded in 100 μl volume in 96-well plates, giving approximately 15.000 293-T cells or 50.000 Jurkat cells per well. Then, 50.000 A3.01-CCR5 T-cells in 100 μl medium were added and the co-culture was incubated at 37°C for 60 h. To restrict free virus infection of the target cells, DEAE dextran was omitted from the culture medium. The extent of cell-cell transmission from the donor to the A3.01-CCR5 target cells was quantified by Gaussia luciferase read-out. (ii) To assess free virus infection, the virus-containing supernatant of the second 12-well of donor cells was harvested and subsequently titrated on either A3.01-CCR5 or TZM-bl target cells in 96-well plates. Assay conditions and free virus input (starting at 100 μl) were chosen to reflect cell-cell transmission co-culture conditions. The extent of free virus infectivity of the target cells was quantified by Gaussia luciferase read-out. Additional file 2:
**Normalization approach for free virus infection and cell-cell transmission data.** **(A)** In both free virus infection and cell-cell transmission setups Gaussia luciferase (GLuc) activity is measured to monitor infection. Raw values are obtained as Relative Light Units (RLU). Let us assume that a wt and a mutant env have RLU values in free virus infection of *a* and *b*, respectively. Similarly, in cell-cell transmission these envs will have RLU values of *c* and *d*, respectively. **(B)** The relative infectivity of the mutant env to the wt env in free virus infection is then given by *a* divided by *b*, multiplied by 100 to obtain the value in percent of wt infectivity. Likewise, the relative infectivity of the mutant env in cell-cell transmission is determined. **(C)** To obtain a measure of the relative efficacy of the mutant env in the two transmission pathways, we divide the relative cell-cell transmission activity by the relative free virus infection capacity. Thus, a value of 1 will indicate equal free virus and cell-cell transmission capacities, a value below 1 indicates better free virus infectivity than cell-cell transmission capacity, and a value greater than 1 indicates better cell-cell transmission capacity than free virus infection capacity. Additional file 3:
**Gaussia luciferase activity of V1V2-deleted and wt envs in free virus infection and cell-cell transmission.**
Additional file 4:
**Correlation analyses of V1V2-deleted env entry and trimer stability characteristics.** **(A)** to **(G)**: All correlations were performed in GraphPad PRISM according to Pearson. **(A)** Correlation analysis of ΔV1V2 env free virus entry fitness and fusion fitness. Data are derived from Figure 3B and C. **(B)** Correlation analysis of ΔV1V2 env free virus entry fitness and virus half-life. Data are derived from Figure 3B and 4B. **(C)** Correlation analysis of ΔV1V2 env free virus entry fitness and temperature sensitivity. Data are derived from Figure 3B and 4C. **(D)** Correlation analysis of ΔV1V2 env fusion fitness and virus half-life. Data are derived from Figure 3C and 4B. **(E)** Correlation analysis of ΔV1V2 env fusion fitness and temperature sensitivity. Data are derived from Figure 3C and 4C. **(F)** Correlation analysis of ΔV1V2 env cell-cell transmission fitness and virus half-life. Data are derived from Figure 3B and 4B. **(G)** Correlation analysis of ΔV1V2 env cell-cell transmission fitness and temperature sensitivity. Data are derived from Figure 3B and 4C. Additional file 5:
**JR-FL wt and ΔV1V2 env expression on transfected 293-T cells and on virions.** **(A)** Example of a flow cytometry histogram of JR-FL wt and ΔV1V2 expressing 293-T cells. Env expression on 293-T cells was detected with biotinylated mAb 2G12 and streptavidin-APC. Mock-transfected cells subjected to the same staining protocol are shown in grey. **(B)** The mean fluorescence intensities (MFI) of 293-T cell populations expressing wt and V1V2-deleted envs and stained with mAb 2G12, including strains JR-FL, NL4-3 and SF162, were calculated. Mean and SD of two independent experiments are shown. **(C)** To derive estimates of average virion gp120 content, pseudotype virus stocks of wt and V1V2-deleted envs of the indicated strains were purified by ultracentrifugation. Subsequently, the purified virus was subjected to gp120 and p24 ELISA. Shown here are the relative gp120 and p24 contents of the V1V2-deleted strains, normalized to each matching wt strain. The p24 content of V1V2-deleted virus stocks was in the same range as the wt stocks, indicating that V1V2 deletion did not affect overall levels of pseudoparticle production. Concerning gp120 content, we observed marked reductions in gp120 content for strains ZM109 ΔV1V2 (approximately 10% of wt gp120 content), which was not functional as free virus, and JR-FL ΔV1V2 (approximately 40% of wt gp120 content). All other strains showed gp120 levels at ≥ 80% of wt. Symbols depict mean values derived from 3 independent experiments with ELISAs performed in duplicates. Additional file 6:
**Long-term incubation infectivity decay curves of wt and V1V2-deleted virions.** Long-term incubation infectivity decay curves for wt virions (black) and V1V2-deleted virions (red). The data shown here were employed to calculate virus half-life as depicted in Figure 4B. Data points are mean and SD from two to three independent experiments performed in duplicates. Additional file 7:
**Temperature gradient infectivity decay curves of wt and V1V2-deleted virions.** Temperature gradient infectivity decay curves for wt virions (black) and V1V2-deleted virions (red). The data shown here were employed to calculate the temperature at which 50% virus infectivity remain as depicted in Figure 4C. Data points are mean and SD from two to three independent experiments performed in duplicates. Additional file 8:
**Correlation analysis of virus half-life and temperature tolerance.** Correlation analyses (according to Pearson) between virus half-life (Figure 4B and Additional file 6) and temperature tolerance (Figure 4C and Additional file 7) are shown for wt virions (top) and V1V2-deleted virions (bottom). Additional file 9:
**Correlation and cell-cell fusion analysis of the JR-CSF env mutant panel.** **(A)** Correlation analysis (according to Pearson) of the data shown in Figure 6. Mutants with high discrepancy between free virus infection and cell-cell transmission capacities are marked in red. **(B)** Correlation analysis upon exclusion of mutants marked red in **(A)**. **(C)** Comparison of free virus infectivity (black) and cell-cell fusion capacity (green) of the JR-CSF mutant panel. Fusion activity followed the same trend as seen for cell-cell transmission (Figure 6) with several mutants losing free virus infection potential but retaining cell-cell fusion capacity. Values of relative efficacy of cell-cell fusion versus free virus infection are shown below the bars; a star indicates whether this difference is statistically significant as probed by multiple unpaired t-tests with alpha = 0.05. Data shown are mean and SD from 3 independent experiments performed in duplicates. Additional file 10:
**Cell-cell fusion analysis of the env N160K/K160N mutant panel.** Analysis of free virus infectivity (black) and cell-cell fusion capacity (green) of the env N160K/K160N mutant panel. We observed similar trends as shown for free virus infection and cell-cell transmission in Figure 7. Envs with the N160K mutation lost in free virus infectivity to varying extent, while cell-cell fusion activity was largely retained. SF162 and Cap88 profited from the K160N mutation and showed enhanced free virus infectivity, while cell-cell fusion capacity was only moderately increased. Values of relative efficacy of cell-cell fusion versus free virus infection are shown below the bars; a star indicates whether this difference is statistically significant as probed by multiple unpaired t-tests with alpha = 0.05. Data shown are mean and SD from 3 independent experiments performed in duplicates. Additional file 11:
**Sequence alignment of SF162 and P3N envs.** Sequences were derived from in-house sequencing of the respective env clones. The V1V2 domain is boxed in red. Residue numbering is based on SF162. In addition to sequence changes in V1V2, several additional residue changes in both gp120 and gp41 are apparent in P3N compared to the parental SF162 env. Additional file 12:
**Correlation analysis of neutralization sensitivity and entry fitness of the SF162/P3N env panel.** Correlation analysis (according to Pearson) of IC50s for mAbs b6 and 1.79 and the free virus entry fitness of the 8 envs of the SF162/P3N V1V2 swap and point mutant panel (neutralization and free virus entry data are shown in Figure 8) highlights considerable correlation between free virus entry capacity and neutralization resistance. Additional file 13:
**A putative model for V1V2 function in free virus infection and cell-cell transmission.** **(A)** In a situation where no neutralizing Abs (black) are present, HIV-1 efficiently infects target cells both as free virus and via cell-cell transmission. **(B)** If neutralizing antibodies (blue) develop that bind and neutralize the contemporaneous virus trimer, free virus infection will be neutralized. However cell-cell transmission, being more resistant to antibody neutralization, may persist at lower levels. **(C)** Eventually, virus escape mutations to the neutralizing antibodies will emerge. These escape mutations may be located in V1V2 (indicated as red V1V2 loops). The escape mutations may interfere with V1V2 function thereby reducing free virus entry capacity (dashed arrow to target cell). However, the mutations may be better tolerated in the context of cell-cell transmission, resulting in preferential virus spread via this transmission pathway (bottom). **(D)** After some rounds of virus replication via cell-cell transmission, mutations in gp120/gp41 may emerge (indicated in yellow) which compensate the defects in V1V2, thereby restoring trimer functionality and free virus infectivity while maintaining antibody resistance.
