# Supplementary material for: Partial rescue of V1V2 mutant infectivity by HIV-1 cell-cell transmission supports the domain’s exceptional capacity for sequence variation
Source: Retrovirology. 2014 Sep 25;11:75. doi: 10.1186/s12977-014-0075-y (PMC4190450; doi:10.1186/s12977-014-0075-y)

Additional File 1

A Assay setups to dissect free virus infection and cell-cell transmission

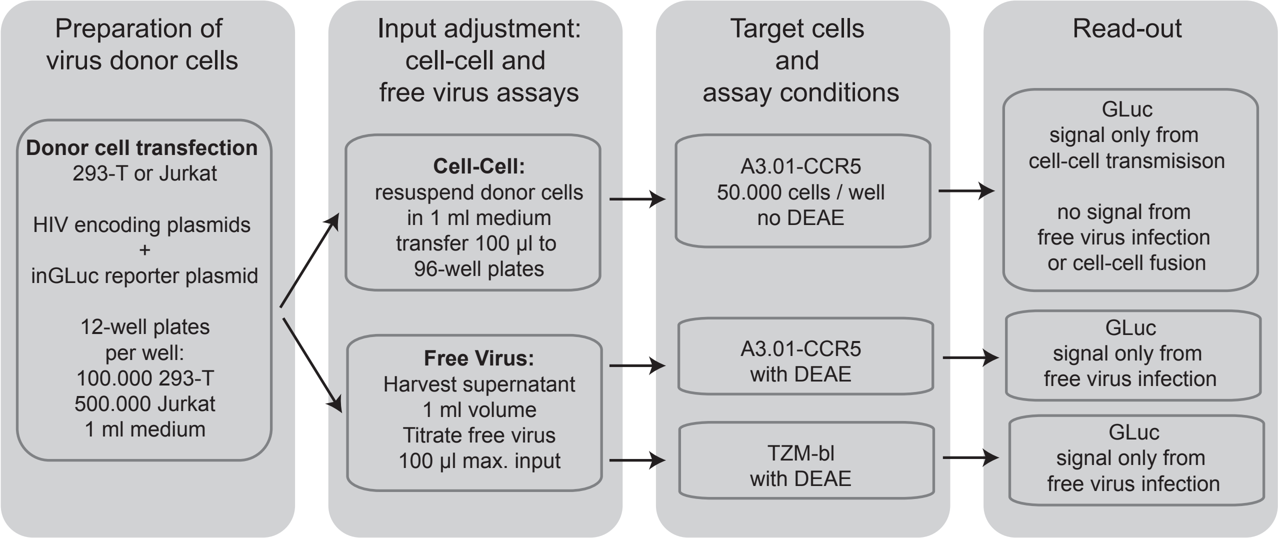

B Assay setup scheme

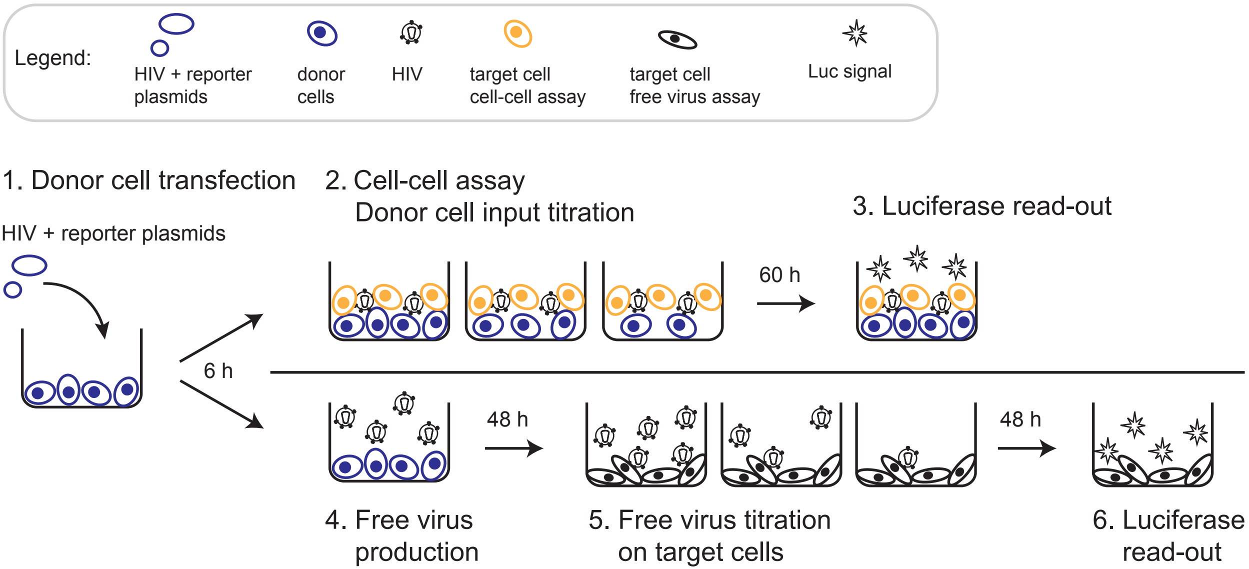

Supplement: Additional file 1: — Assay setups to study free virus infection and cell-cell transmission. (A) Overview of the assay formats used to dissect free virus infection and cell-cell transmission. (B) Experimental assay scheme: To study and quantitatively compare HIV free virus infection and cell-cell transmission virus donor cells (either 293-T cells or Jurkat T cells) were transfected with HIV encoding plasmids and the inGLuc reporter plasmid. Transfection was performed in 12-well plates, with two wells per env. Six hours post-transfection, one well was processed for cell-cell transmission, and one well for free virus infection: (i) To assess cell-cell transmission, the donor cells were resuspended in 1 ml medium and re-seeded in 100 μl volume in 96-well plates, giving approximately 15.000 293-T cells or 50.000 Jurkat cells per well. Then, 50.000 A3.01-CCR5 T-cells in 100 μl medium were added and the co-culture was incubated at 37°C for 60 h. To restrict free virus infection of the target cells, DEAE dextran was omitted from the culture medium. The extent of cell-cell transmission from the donor to the A3.01-CCR5 target cells was quantified by Gaussia luciferase read-out. (ii) To assess free virus infection, the virus-containing supernatant of the second 12-well of donor cells was harvested and subsequently titrated on either A3.01-CCR5 or TZM-bl target cells in 96-well plates. Assay conditions and free virus input (starting at 100 μl) were chosen to reflect cell-cell transmission co-culture conditions. The extent of free virus infectivity of the target cells was quantified by Gaussia luciferase read-out. [file 12977_2014_75_MOESM1_ESM.pdf]
