# Supplementary material for: Partial rescue of V1V2 mutant infectivity by HIV-1 cell-cell transmission supports the domain’s exceptional capacity for sequence variation
Source: Retrovirology. 2014 Sep 25;11:75. doi: 10.1186/s12977-014-0075-y (PMC4190450; doi:10.1186/s12977-014-0075-y)
Supplement: Additional file 2: — Normalization approach for free virus infection and cell-cell transmission data. (A) In both free virus infection and cell-cell transmission setups Gaussia luciferase (GLuc) activity is measured to monitor infection. Raw values are obtained as Relative Light Units (RLU). Let us assume that a wt and a mutant env have RLU values in free virus infection of a and b, respectively. Similarly, in cell-cell transmission these envs will have RLU values of c and d, respectively. (B) The relative infectivity of the mutant env to the wt env in free virus infection is then given by a divided by b, multiplied by 100 to obtain the value in percent of wt infectivity. Likewise, the relative infectivity of the mutant env in cell-cell transmission is determined. (C) To obtain a measure of the relative efficacy of the mutant env in the two transmission pathways, we divide the relative cell-cell transmission activity by the relative free virus infection capacity. Thus, a value of 1 will indicate equal free virus and cell-cell transmission capacities, a value below 1 indicates better free virus infectivity than cell-cell transmission capacity, and a value greater than 1 indicates better cell-cell transmission capacity than free virus infection capacity. [file 12977_2014_75_MOESM2_ESM.pdf]

Additional File 2

Derivation of relative infectivities free virus infection and cell-cell transmission

A

Raw Data (in Relative Light Units, RLU):

	free virus infection	cell-cell transmission
wt env	***a***	***c***
mutant env	***b***	***d***

B

Normalization mutant to wt env infectivity:

Free virus infection:
% mutant infectivity (***x***) = $\frac{b}{a}$ x 100

Cell-cell transmission:
% mutant infectivity (***y***) = $\frac{d}{c}$ x 100

C

Relative cell-cell to free virus infection efficacy of mutant env:

Fold difference cell-cell transmission to free virus infectivity = $\frac{y}{x}$
