# Supplementary material for: Partial rescue of V1V2 mutant infectivity by HIV-1 cell-cell transmission supports the domain’s exceptional capacity for sequence variation
Source: Retrovirology. 2014 Sep 25;11:75. doi: 10.1186/s12977-014-0075-y (PMC4190450; doi:10.1186/s12977-014-0075-y)
Supplement: Additional file 3: — Gaussia luciferase activity of V1V2-deleted and wt envs in free virus infection and cell-cell transmission. [file 12977_2014_75_MOESM3_ESM.pdf]

Additional File 3

Gaussia luciferase activity of V1V2-deleted and wt envs in free virus infection and cell-cell transmission [a]

Strain	Type	free virus infection [b] TZM-bl RLU (Gaussia Luciferase)	cell-cell transmission [c] 293-T : A3.01-CCR5 RLU (Gaussia Luciferase)
SF162	wt	430200	45409
	ΔV1V2	442100	44325
NL4-3	wt	61800	20361
	ΔV1V2	14200	1978
JR-FL	wt	666000	28620
	ΔV1V2	7700	2374
RHPA	wt	630900	21816
	ΔV1V2	6000	722
AC10	wt	282300	15664
	ΔV1V2	13300	2998
REJO	wt	520300	35187
	ΔV1V2	32600	3416
ZA110	wt	868200	27427
	ΔV1V2	82900	5866
CAP88	wt	70600	2167
	ΔV1V2	2900	533
ZM109	wt	75300	4410
	ΔV1V2	1100	524
ZM214	wt	40900	23003
	ΔV1V2	7800	19185

[a] Relative light units (RLUs) derived for experiments depicted in Figure 3B. The RLUs shown for free virus infection and cell-cell transmission correspond to 100 µl infectious input derived from the same 293-T donor cells.

[b] To determine free virus infectivity virus supernatant from the 293-T donor cells was harvested and titrated on TZM-bl cells to determine Gaussia Luciferase activity per 100 µl virus input. Values are means of 3 independent experiments with virus titrations performed in duplicates.

[c] To determine cell-cell transmission 293-T donor cells were harvested and 100 µl aliquots (corresponding to 15.000 293-T donor cells) were used for co-culture with 50.000 A3.01-CCR5 cells in 96-wells. Values are means of 3 independent experiments performed with 5 replicates.
