# Supplementary figures and images for: Partial rescue of V1V2 mutant infectivity by HIV-1 cell-cell transmission supports the domain’s exceptional capacity for sequence variation
Source: Retrovirology. 2014 Sep 25;11:75. doi: 10.1186/s12977-014-0075-y (PMC4190450; doi:10.1186/s12977-014-0075-y)

# Additional File 4

**A**

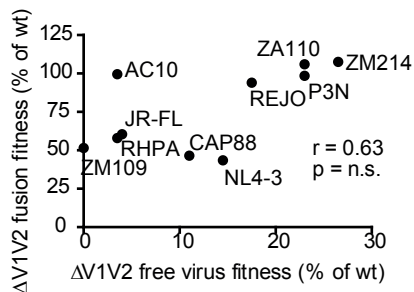

**B**

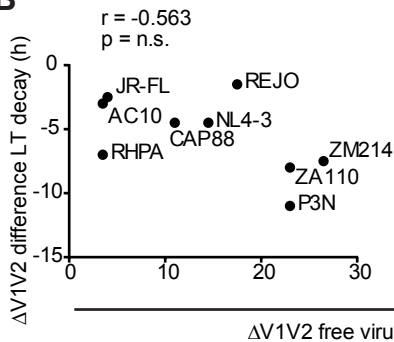

**C**

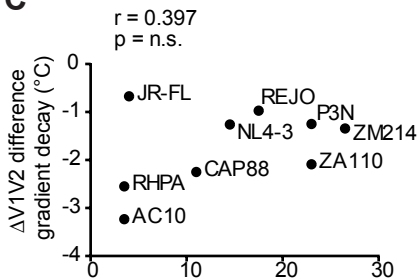

**D**

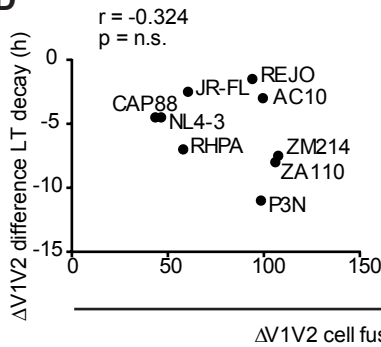

**E**

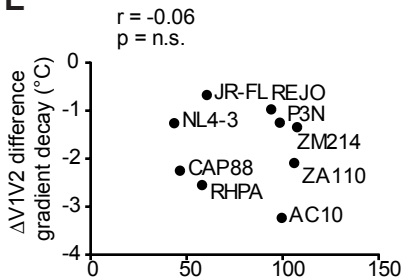

**F**

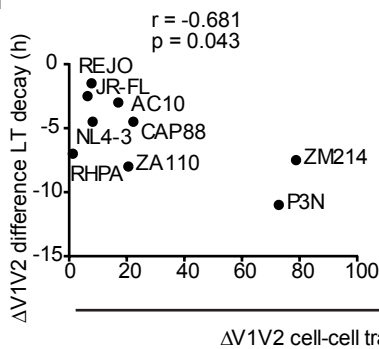

**G**

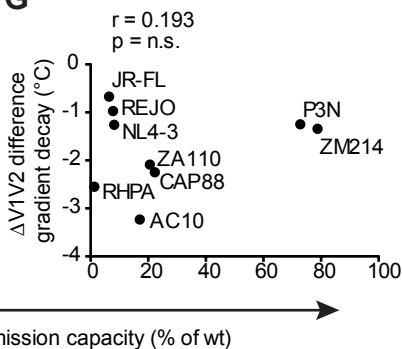

Supplement: Additional file 4: — Correlation analyses of V1V2-deleted env entry and trimer stability characteristics. (A) to (G): All correlations were performed in GraphPad PRISM according to Pearson. (A) Correlation analysis of ΔV1V2 env free virus entry fitness and fusion fitness. Data are derived from Figure 3B and C. (B) Correlation analysis of ΔV1V2 env free virus entry fitness and virus half-life. Data are derived from Figure 3B and 4B. (C) Correlation analysis of ΔV1V2 env free virus entry fitness and temperature sensitivity. Data are derived from Figure 3B and 4C. (D) Correlation analysis of ΔV1V2 env fusion fitness and virus half-life. Data are derived from Figure 3C and 4B. (E) Correlation analysis of ΔV1V2 env fusion fitness and temperature sensitivity. Data are derived from Figure 3C and 4C. (F) Correlation analysis of ΔV1V2 env cell-cell transmission fitness and virus half-life. Data are derived from Figure 3B and 4B. (G) Correlation analysis of ΔV1V2 env cell-cell transmission fitness and temperature sensitivity. Data are derived from Figure 3B and 4C. [file 12977_2014_75_MOESM4_ESM.pdf]

# Additional File 5

**A**

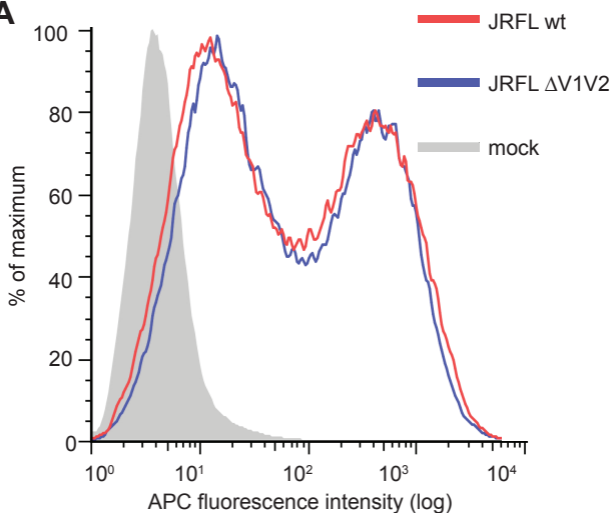

**B**

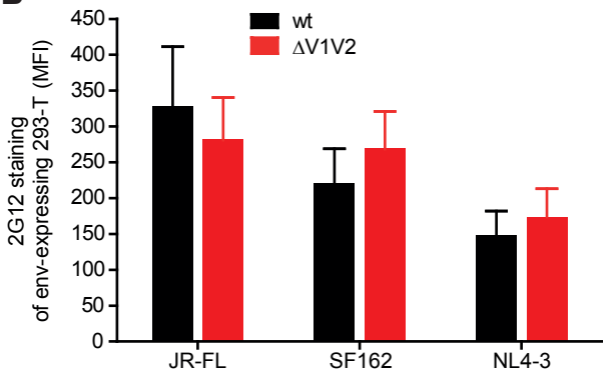

**C**

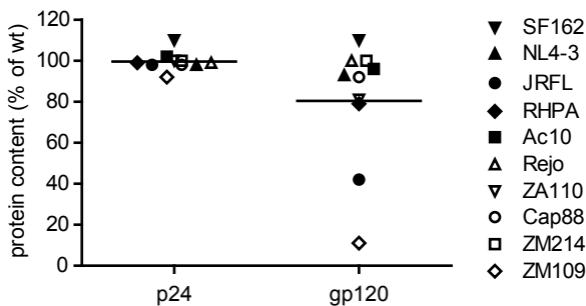

Supplement: Additional file 5: — JR-FL wt and ΔV1V2 env expression on transfected 293-T cells and on virions. (A) Example of a flow cytometry histogram of JR-FL wt and ΔV1V2 expressing 293-T cells. Env expression on 293-T cells was detected with biotinylated mAb 2G12 and streptavidin-APC. Mock-transfected cells subjected to the same staining protocol are shown in grey. (B) The mean fluorescence intensities (MFI) of 293-T cell populations expressing wt and V1V2-deleted envs and stained with mAb 2G12, including strains JR-FL, NL4-3 and SF162, were calculated. Mean and SD of two independent experiments are shown. (C) To derive estimates of average virion gp120 content, pseudotype virus stocks of wt and V1V2-deleted envs of the indicated strains were purified by ultracentrifugation. Subsequently, the purified virus was subjected to gp120 and p24 ELISA. Shown here are the relative gp120 and p24 contents of the V1V2-deleted strains, normalized to each matching wt strain. The p24 content of V1V2-deleted virus stocks was in the same range as the wt stocks, indicating that V1V2 deletion did not affect overall levels of pseudoparticle production. Concerning gp120 content, we observed marked reductions in gp120 content for strains ZM109 ΔV1V2 (approximately 10% of wt gp120 content), which was not functional as free virus, and JR-FL ΔV1V2 (approximately 40% of wt gp120 content). All other strains showed gp120 levels at ≥ 80% of wt. Symbols depict mean values derived from 3 independent experiments with ELISAs performed in duplicates. [file 12977_2014_75_MOESM5_ESM.pdf]

# Additional File 6

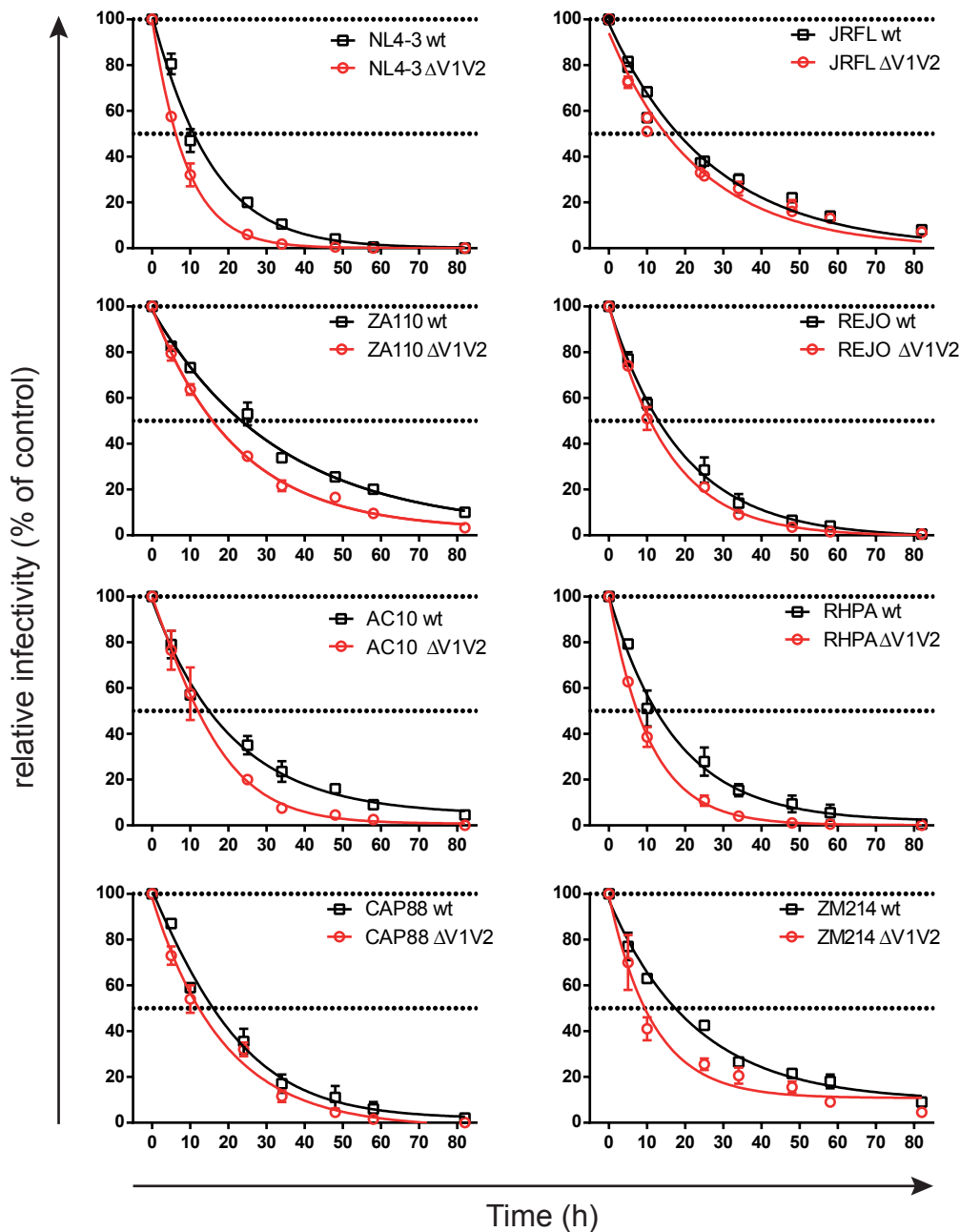

Supplement: Additional file 6: — Long-term incubation infectivity decay curves of wt and V1V2-deleted virions. Long-term incubation infectivity decay curves for wt virions (black) and V1V2-deleted virions (red). The data shown here were employed to calculate virus half-life as depicted in Figure 4B. Data points are mean and SD from two to three independent experiments performed in duplicates. [file 12977_2014_75_MOESM6_ESM.pdf]

# Additional File 7

relative infectivity (% of control)

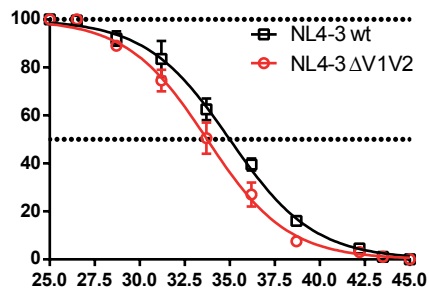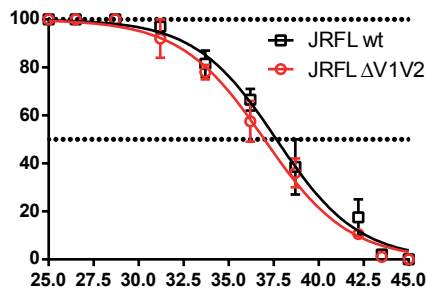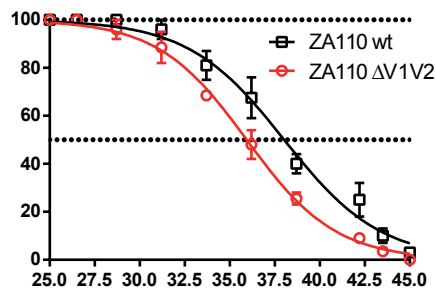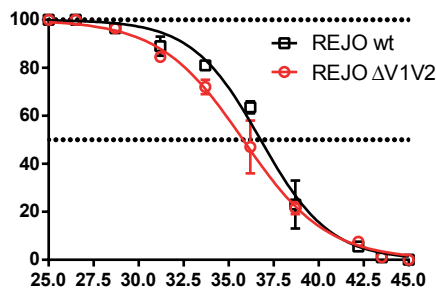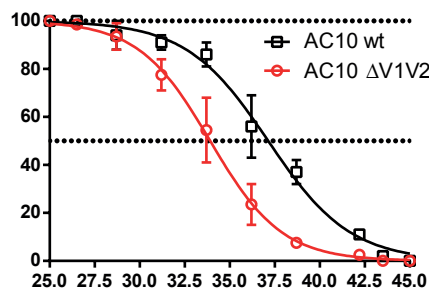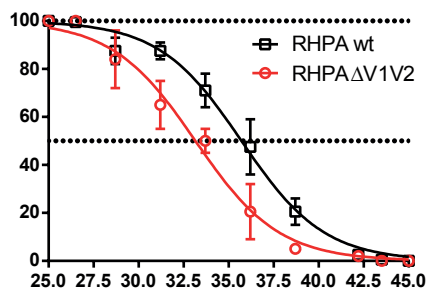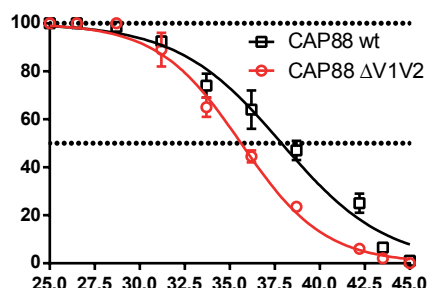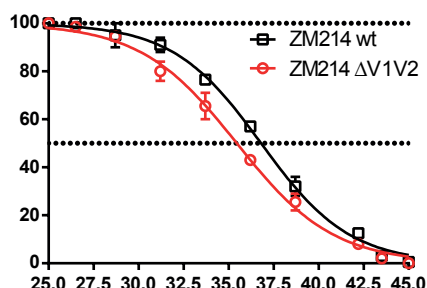

Temperature (°C)

Supplement: Additional file 7: — Temperature gradient infectivity decay curves of wt and V1V2-deleted virions. Temperature gradient infectivity decay curves for wt virions (black) and V1V2-deleted virions (red). The data shown here were employed to calculate the temperature at which 50% virus infectivity remain as depicted in Figure 4C. Data points are mean and SD from two to three independent experiments performed in duplicates. [file 12977_2014_75_MOESM7_ESM.pdf]

## Additional File 8

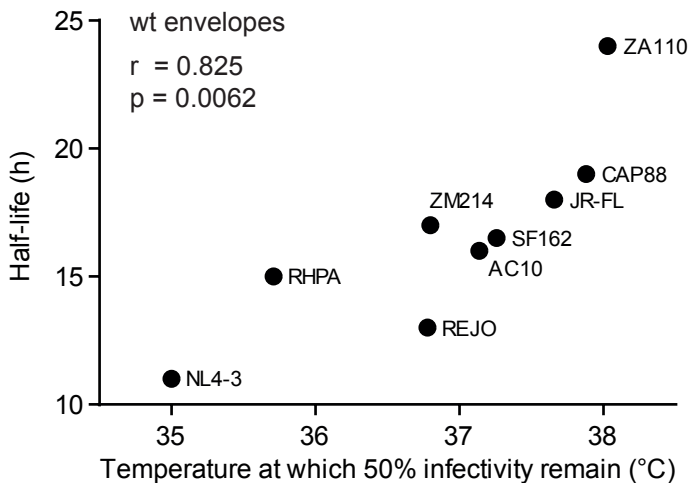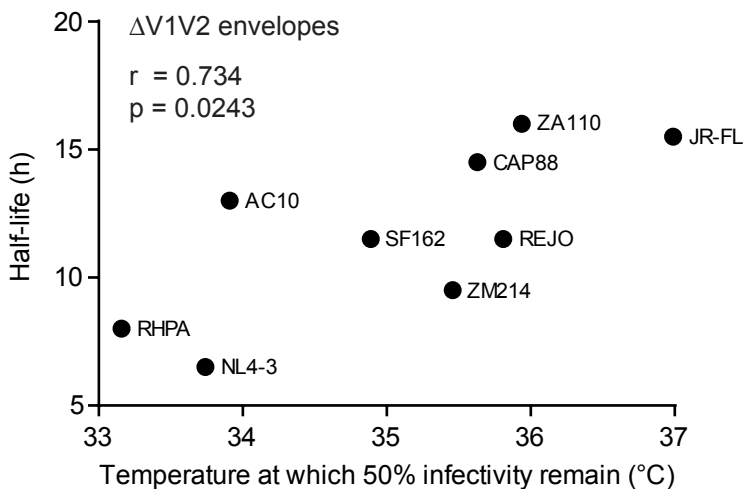

Supplement: Additional file 8: — Correlation analysis of virus half-life and temperature tolerance. Correlation analyses (according to Pearson) between virus half-life (Figure 4B and Additional file 6) and temperature tolerance (Figure 4C and Additional file 7) are shown for wt virions (top) and V1V2-deleted virions (bottom). [file 12977_2014_75_MOESM8_ESM.pdf]

# Additional File 9

**A**

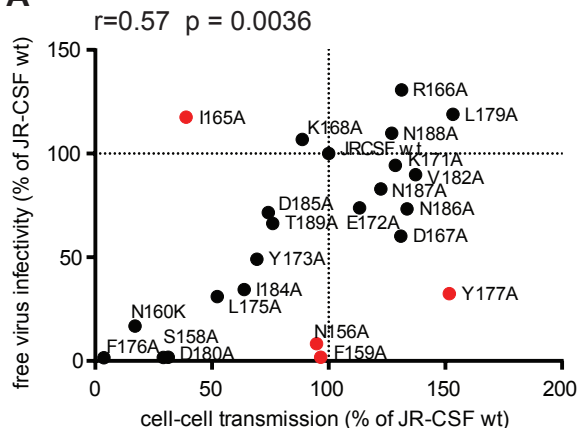

**B**

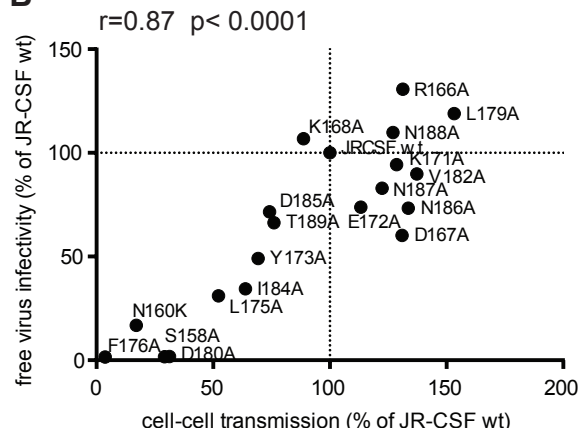

**C**

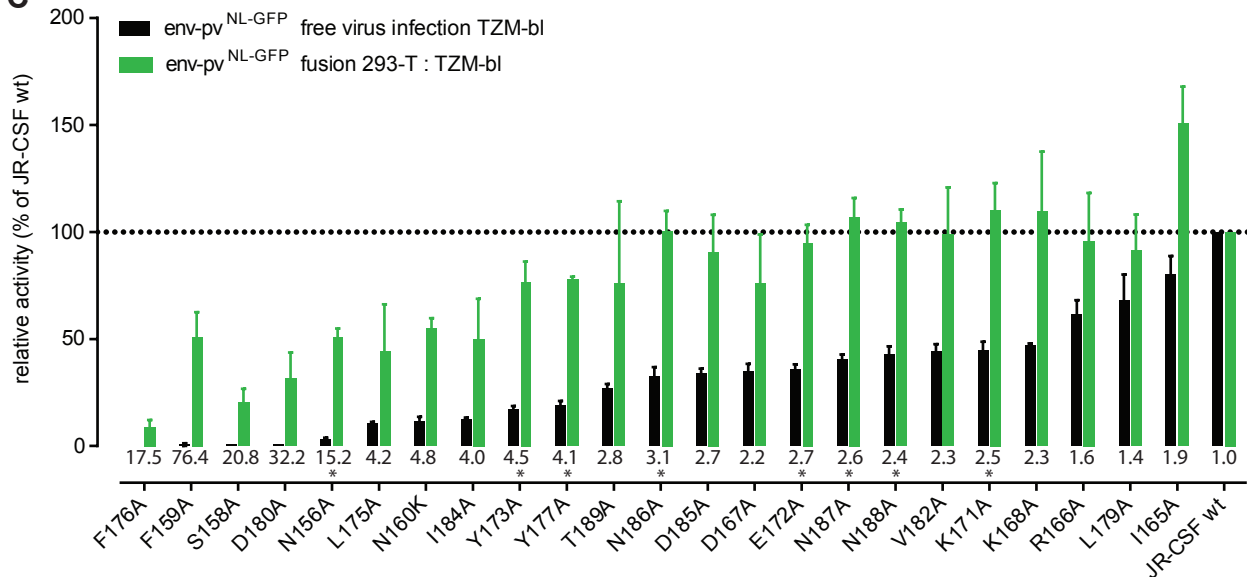

Supplement: Additional file 9: — Correlation and cell-cell fusion analysis of the JR-CSF env mutant panel. (A) Correlation analysis (according to Pearson) of the data shown in Figure 6. Mutants with high discrepancy between free virus infection and cell-cell transmission capacities are marked in red. (B) Correlation analysis upon exclusion of mutants marked red in (A). (C) Comparison of free virus infectivity (black) and cell-cell fusion capacity (green) of the JR-CSF mutant panel. Fusion activity followed the same trend as seen for cell-cell transmission (Figure 6) with several mutants losing free virus infection potential but retaining cell-cell fusion capacity. Values of relative efficacy of cell-cell fusion versus free virus infection are shown below the bars; a star indicates whether this difference is statistically significant as probed by multiple unpaired t-tests with alpha = 0.05. Data shown are mean and SD from 3 independent experiments performed in duplicates. [file 12977_2014_75_MOESM9_ESM.pdf]

# Additional File 10

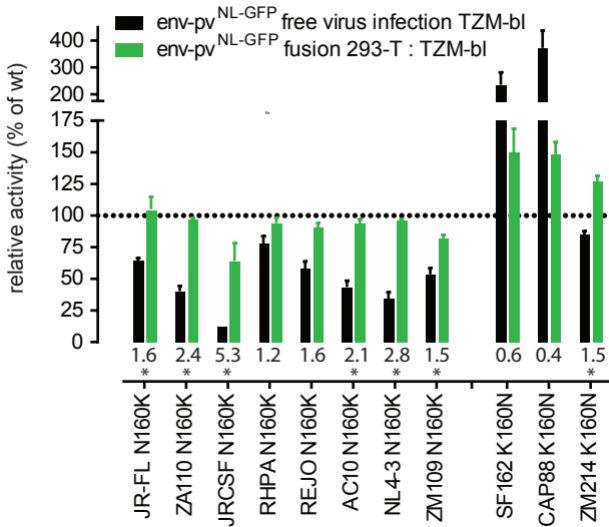

Supplement: Additional file 10: — Cell-cell fusion analysis of the env N160K/K160N mutant panel. Analysis of free virus infectivity (black) and cell-cell fusion capacity (green) of the env N160K/K160N mutant panel. We observed similar trends as shown for free virus infection and cell-cell transmission in Figure 7. Envs with the N160K mutation lost in free virus infectivity to varying extent, while cell-cell fusion activity was largely retained. SF162 and Cap88 profited from the K160N mutation and showed enhanced free virus infectivity, while cell-cell fusion capacity was only moderately increased. Values of relative efficacy of cell-cell fusion versus free virus infection are shown below the bars; a star indicates whether this difference is statistically significant as probed by multiple unpaired t-tests with alpha = 0.05. Data shown are mean and SD from 3 independent experiments performed in duplicates. [file 12977_2014_75_MOESM10_ESM.pdf]

Additional File 11

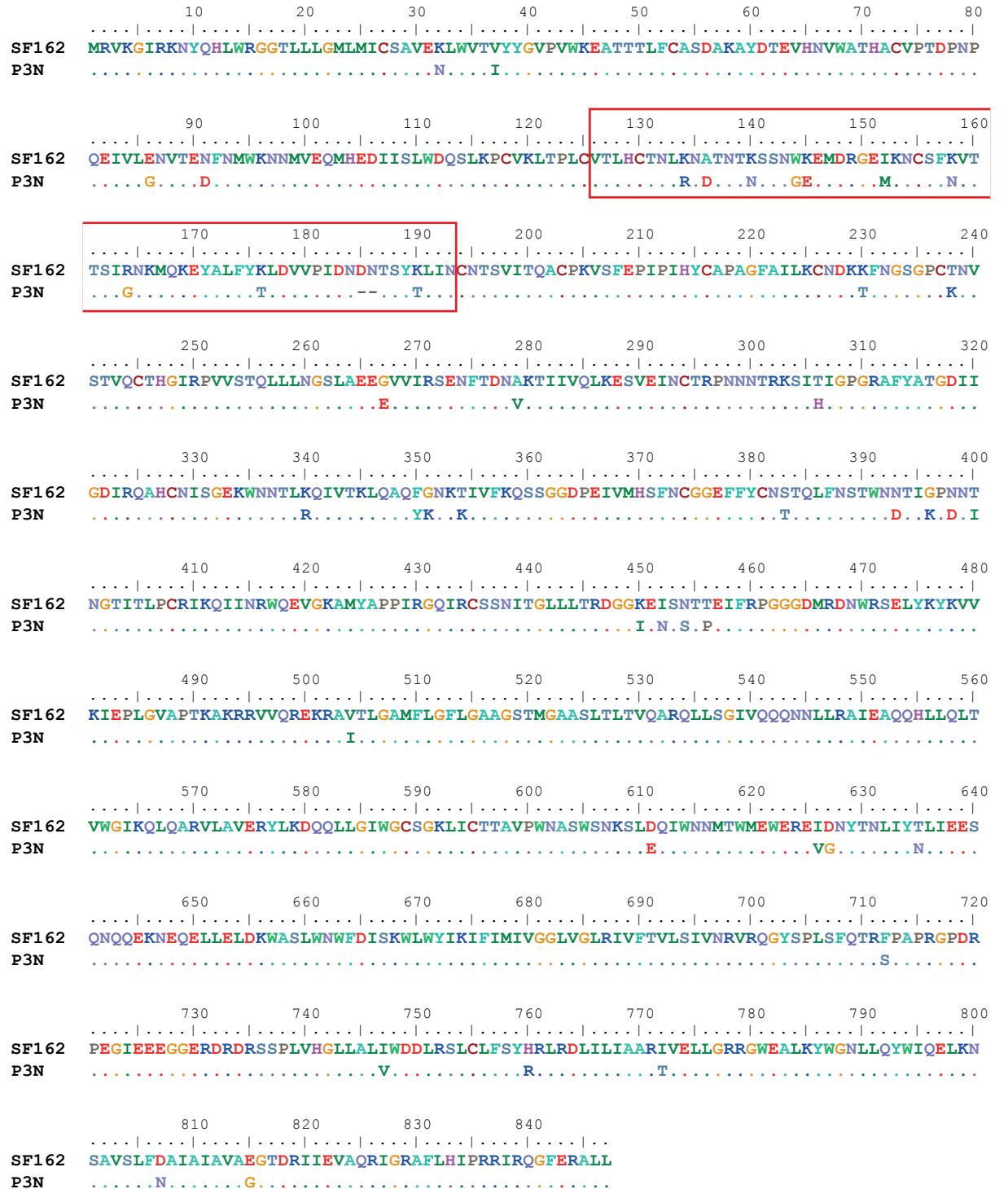

Supplement: Additional file 11: — Sequence alignment of SF162 and P3N envs. Sequences were derived from in-house sequencing of the respective env clones. The V1V2 domain is boxed in red. Residue numbering is based on SF162. In addition to sequence changes in V1V2, several additional residue changes in both gp120 and gp41 are apparent in P3N compared to the parental SF162 env. [file 12977_2014_75_MOESM11_ESM.pdf]
