# Supplementary material for: Partial rescue of V1V2 mutant infectivity by HIV-1 cell-cell transmission supports the domain’s exceptional capacity for sequence variation
Source: Retrovirology. 2014 Sep 25;11:75. doi: 10.1186/s12977-014-0075-y (PMC4190450; doi:10.1186/s12977-014-0075-y)
Supplement: Additional file 12: — Correlation analysis of neutralization sensitivity and entry fitness of the SF162/P3N env panel. Correlation analysis (according to Pearson) of IC50s for mAbs b6 and 1.79 and the free virus entry fitness of the 8 envs of the SF162/P3N V1V2 swap and point mutant panel (neutralization and free virus entry data are shown in Figure 8) highlights considerable correlation between free virus entry capacity and neutralization resistance. [file 12977_2014_75_MOESM12_ESM.pdf]

Additional File 12

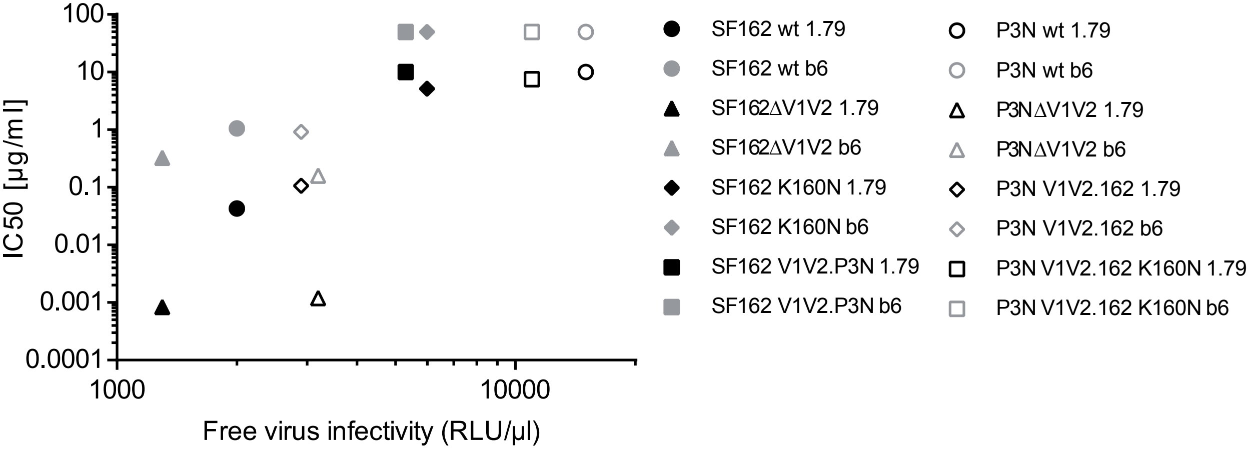

Correlation analysis:

IC50 b6 vs. infectivity: $r = 0.809$, $p = 0.015$

IC50 1.79 vs. infectivity: $r = 0.775$, $p = 0.0238$
