# Supplementary material for: Partial rescue of V1V2 mutant infectivity by HIV-1 cell-cell transmission supports the domain’s exceptional capacity for sequence variation
Source: Retrovirology. 2014 Sep 25;11:75. doi: 10.1186/s12977-014-0075-y (PMC4190450; doi:10.1186/s12977-014-0075-y)

Additional File 13

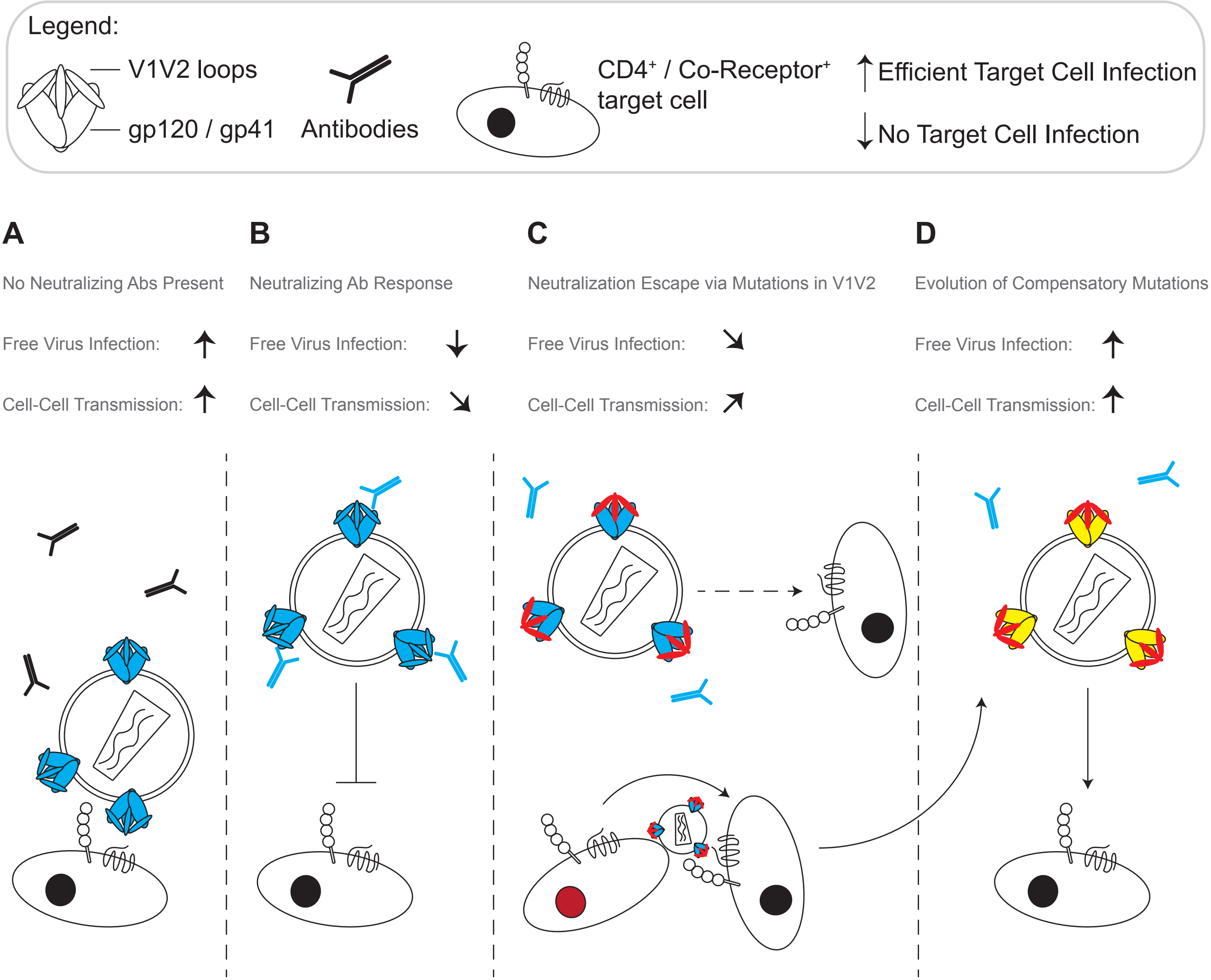

Supplement: Additional file 13: — A putative model for V1V2 function in free virus infection and cell-cell transmission. (A) In a situation where no neutralizing Abs (black) are present, HIV-1 efficiently infects target cells both as free virus and via cell-cell transmission. (B) If neutralizing antibodies (blue) develop that bind and neutralize the contemporaneous virus trimer, free virus infection will be neutralized. However cell-cell transmission, being more resistant to antibody neutralization, may persist at lower levels. (C) Eventually, virus escape mutations to the neutralizing antibodies will emerge. These escape mutations may be located in V1V2 (indicated as red V1V2 loops). The escape mutations may interfere with V1V2 function thereby reducing free virus entry capacity (dashed arrow to target cell). However, the mutations may be better tolerated in the context of cell-cell transmission, resulting in preferential virus spread via this transmission pathway (bottom). (D) After some rounds of virus replication via cell-cell transmission, mutations in gp120/gp41 may emerge (indicated in yellow) which compensate the defects in V1V2, thereby restoring trimer functionality and free virus infectivity while maintaining antibody resistance. [file 12977_2014_75_MOESM13_ESM.pdf]
